# Non-steroidal anti-inflammatory drug target gene associations with major depressive disorders: a Mendelian randomisation study integrating GWAS, eQTL and mQTL Data

**DOI:** 10.1038/s41397-023-00302-1

**Published:** 2023-03-25

**Authors:** Qian He, Kevin Chun Hei Wu, Adam N. Bennett, Beifang Fan, Jundong Liu, Ruixuan Huang, Alice P. S. Kong, Xiaoyu Tian, Man Ki Maggie Kwok, Kei Hang Katie Chan

**Affiliations:** 1 Department of Biomedical Sciences, City University of Hong Kong, Hong Kong SAR, China; 2 Department of Mental Health, Shenzhen Nanshan Centre for Chronic Disease Control, Shenzhen, China; 3 Department of Electrical Engineering, City University of Hong Kong, Hong Kong SAR, China; 4 Department of Medicine and Therapeutics, The Chinese University of Hong Kong, Hong Kong SAR, China; 5 Hong Kong Institute of Diabetes and Obesity, The Chinese University of Hong Kong, Hong Kong SAR, China; 6 Li Ka Shing Institute of Health Sciences, The Chinese University of Hong Kong, Hong Kong SAR, China; 7 School of Biomedical Sciences and Li Ka Shing Institute of Health Science, The Chinese University of Hong Kong, Hong Kong SAR, China; 8 Heart and Vascular Institute and Shenzhen Research Institute, The Chinese University of Hong Kong, Hong Kong SAR, China; 9 School of Public Health, Li Ka Shing Faculty of Medicine, The University of Hong Kong, Hong Kong SAR, China; 10 Department of Epidemiology, Centre for Global Cardiometabolic Health, Brown University, Providence, RI USA

**Keywords:** Predictive medicine, Human behaviour

## Abstract

Previous observational studies reported associations between non-steroidal anti-inflammatory drugs (NSAIDs) and major depressive disorder (MDD), however, these associations are often inconsistent and underlying biological mechanisms are still poorly understood. We conducted a two-sample Mendelian randomisation (MR) study to examine relationships between genetic variants and NSAID target gene expression or DNA methylation (DNAm) using publicly available expression, methylation quantitative trait loci (eQTL or mQTL) data and genetic variant-disease associations from genome-wide association studies (GWAS of MDD). We also assessed drug exposure using gene expression and DNAm levels of NSAID targets as proxies. Genetic variants were robustly adjusted for multiple comparisons related to gene expression, DNAm was used as MR instrumental variables and GWAS statistics of MDD as the outcome. A 1-standard deviation (SD) lower expression of *NEU1* in blood was related to lower C-reactive protein (CRP) levels of −0.215 mg/L (95% confidence interval (CI): 0.128–0.426) and a decreased risk of MDD (odds ratio [OR] = 0.806; 95% CI: 0.735–0.885; *p* = 5.36 × 10^−6^). A concordant direction of association was also observed for *NEU1* DNAm levels in blood and a risk of MDD (OR = 0.886; 95% CI: 0.836–0.939; *p* = 4.71 × 10^−5^). Further, the genetic variants associated with MDD were mediated by *NEU1* expression via DNAm (*β* = −0.519; 95% CI: −0.717 to −0.320256; *p* = 3.16 × 10^−7^). We did not observe causal relationships between inflammatory genetic marker estimations and MDD risk. Yet, we identified a concordant association of *NEU1* messenger RNA and an adverse direction of association of higher *NEU1* DNAm with MDD risk. These results warrant increased pharmacovigilance and further in vivo or in vitro studies to investigate *NEU1* inhibitors or supplements for MDD.

## Introduction

From the literature, associations between cell inflammation and depression are highly credible [1]. Patients with MDD exhibit increased inflammatory factor levels that are typically related to chronic inflammation, including inflammatory cytokines such as tumor necrosis factor (TNF), interleukin-1β (IL-1β) and interleukin-6 (IL-6) and acute phase proteins such as C-reactive protein (CRP) [2]. Increased inflammatory markers have also been associated with several depressive symptoms and suicidal MDD [3]. A study measuring cytokines in ‘never-treated’ relatively homogeneous patients with MDD reported that the majority of cytokines were elevated [4].

Elevated inflammatory factor levels in individuals with MDD may be attributable to ineffective clinical management [5]. For example, chronic depression and elevated depression severity at follow-up could be predicted by higher IL-6 levels [6]. CRP and TNF have also been associated with symptom severity in MDD patients [7]. Stimulation-like inflammation and infection may occur via activation of the microglia, which are the brain’s immune cells, to secrete proinflammatory cytokines that act on MDD related pathways, such hypothalamic-pituitary-adrenal (HPA) axis activation and increased indoleamine-2,3-deoxygenase activity [8]. Recent evidence has also suggested that persistent low-level inflammation is related to treatment-resistant depression and low responses to antidepressant treatments [8, 9].

Based on these evidence, recent studies examined the effects of modulating the immune system during MDD [9–11]; they used NSAIDs as add-ons to conventional antidepressant therapy, however, data also exists for NSAIDs monotherapy as antidepressants [10–12]. A recent meta-analysis summarised 36 randomised controlled trials (RCTs), including 10,000 patients, and reported that both monotherapy, add-on NSAIDs therapy and other add-on therapies, such as cytokine-inhibitor monotherapy and statin add-on therapy, generated antidepressant efficacy [12]. However, in contrast, previous studies also showed that NSAID efficacy for depressive symptoms was negligible [11, 13].

Over the past 20 years, studies have proposed the notion that inflammation and brain-immune interactions are involved in MDD pathogenesis [14, 15]. However, due to a lack of data consistency in terms of add-ons to conventional anti-depression drugs and high treatment resistance rates, other mechanisms such as genetics are proposed to affect NSAIDs efficacy towards depression. Thus, while many studies have explored the effects of NSAIDs on MDD, the biological mechanisms underpinning NSAIDs function in depressed patients remain poorly understood. Given the higher prevalence of inflammation in patients with MDD, understanding the mechanistic links between NSAIDs and MDD may identify more targeted anti-depressive therapies and facilitate better-informed prescription decisions by clinicians for patients with comorbidity [16].

Typically, RCTs are highly structured investigations used to identify the causal effects of drugs in disease [17]. However, RCTs are expensive and time-consuming, which could explain why there has been a lack of large, high-quality RCTs exploring the effects of NSAIDs in MDD. RCTs typically evaluate the relatively short-term effects of an intervention on intermediate biomarkers or populations, but without estimating the effects on genetic variants [18]. MR uses genetic variants which are robustly associated with exposure as instrumental variables to explore causal associations between the exposure and outcome [18]. Additionally, the effects of genetic variants used to instrument variables in the MR design are present at conception [19], thus MR studies can be used to estimate the long-term effects of exposure of interest on the risk of an outcome [17]. Genetic variants related to NSAIDs target messenger RNA expression or DNA methylation (DNAm), also called expression or methylation quantitative trait loci (eQTLs or mQTLs, respectively), and can be viewed as instrumental variables representing NSAIDs exposure [20, 21]. In this study, we used eQTL and mQTL data to identify suitable genetic instruments to investigate the expression of NSAIDs targets. Using this strategy, MR analysis could overcome the limitations of observational studies, e.g., by limiting potential confounders, inferring causality and using existing study samples.

Recent studies investigated CRP [21] and IL-6 [22] associations with MDD risk and suggested a potential causal relationship between inflammatory factors and a subset of depressive symptoms. However, it is unknown if NSAIDs affect MDD and if these putative affects towards MDD are mediated by inflammatory factors.

In this study, we sought to identify NSAIDs associations with MDD using a two-sample MR approach and multi-omics datasets. We used publicly available eQTL and mQTL datasets to identify suitable genetic instruments for gene expression and DNAm (drug exposure proxies) analyses of NSAIDs targets. We also used the most recent and largest MDD GWAS to explore and characterise associations between NSAIDs, inflammatory factors and MDD.

## Subjects and methods

### Identifying NSAID target genes

We identified different NSAIDs classes using the Anatomical Therapeutic Chemical classification system using the World Health Organisation Collaborating Centre for Drug Statistics Methodology. Genes whose protein products were targeted by any of NSAIDs active ingredients were identified using DrugBank [23] (https://go.drugbank.com/) and ChEMBL [24] (https://www.ebi.ac.uk/chembl/) databases. We only included genes that were named as NASIDs targets in both databases.

### Genetic instruments for NSAID target gene expression

Using a publicly available dataset from the eQTLGen consortium [25], we identified genetic variants with a minor allele frequency > 0.01 which were related to the expression of NSAIDs targets (*n* = 31,684). Only *cis* regions were accessed with a distance of single nucleotide polymorphisms (SNPs). The eQTL dataset was limited to a 1-SD change in gene expression levels for each affected allele. The *cis* region was defined as within 2 Mb of a probe in either direction. We used Consortium for the Architecture of Gene Expression (CAGE) eQTL summary data [26] (*n* = 2765; predominantly European heritage) to validate genetic instrument of inflammation identification (eTable 1, Supplementary Tables).

We explored DNAm levels of significant NSAIDs target genes in blood using a meta-analysis of blood mQTL [27] data from the Brisbane Systems Genetics Study (*n* = 614) and the Lothian Birth Cohorts of 1921 and 1936 (*n* = 1366). Participants were all of European ancestry (eTable 1, Supplementary Tables). We annotated the closest genes to DNAm probes using annotation files from Price et al. [28]. We also used whole blood mQTL data from the UKHLS study (*n* = 1193; European heritage) to validate our results [29].

### Outcome data

Publicly available GWAS summary data for MDD (170,756 cases and 329,443 controls of European ancestry) were downloaded from the website, and excluded UK Biobank participants [30] (eTable 2, Supplementary Tables). Moreover, we also used GWAS MDD summary data from the UK Biobank (UKB) (113,769 cases and 208,811 controls of European ancestry) to validate our analyses [31] (eTable 2, Supplementary Tables).

### Using summary-based MR (SMR) and independent heterogeneity instruments (HEIDI) to detect pleiotropic associations

#### MR analysis of NSAIDs target gene expression in blood and inflammation

We used the SMR method [32] to perform two-sample MR analyses (Supplementary Methods). To identify associations between changes in gene expression and inflammatory factor levels with drug exposure, SMR analyses were conducted using blood gene expression as the exposure and inflammatory factor levels as the outcome. Inflammatory factor levels were assessed using GWAS summary data for CRP [33] (*n* = 204,402), TNF [34] (*n* = 21,758), IL-6 [35] (*n* = 1301) and IL-1β [36] (*n* = 4910) in European ancestry (eTable 2, Supplementary Tables). We only included potential genes whose expression in blood was related to inflammatory factor levels at a nominal *p* value (*p* < 0.05) in analyses. Estimates from SMR for assessing associations between gene expression and inflammatory factors represented changes in inflammatory factor levels per 1-SD increase in gene expression.

#### MR analysis of NSAIDs target gene expression and DNAm in blood and MDD

SMR analyses were performed to estimate associations of a 1-SD change in gene expression (eQTL data) and DNAm (mQTL data) in MDD (GWAS summary data). Main results were presented as binary variables, such as OR for MDD per 1-SD change in gene and DNAm expression, where the direction of expression for the gene or DNAm change was harmonised to show inflammatory factor level decreasing associations. To control the genome-wide type I error rate, Bonferroni corrections were used to account for multiple testing.

#### MR analysis integrate multi-omics data in blood

We conducted an SMR analysis using blood DNAm as the exposure and gene expression as the outcome. This was to evaluate the relationship of a 1-SD change in DNAm with transcript levels, which were significantly associated with inflammatory factor levels and MDD. For SMR and HEIDI evaluations of associations between DNAm and transcripts, we only tested for relationships between single DNAm sites and single gene expression probes within a 2 Mb distance of *cis* regions. Bonferroni correlations were used to adjust for multiple comparisons.

### Two-sample MR analyses evaluating associations between inflammatory factor levels and MDD

We evaluated association estimates between inflammatory factor levels and MDD using the generalised summary data-based MR (GSMR) method—we used multiple genetic variants, which were related to risk factors, as instruments to identify potential causality [37]. Genome-wide Complex Trait Analysis (version 1.91.7β1) was used to perform GSMR. HEIDI excludes linkage SNPs (i.e., two distant genetic variants in linkage disequilibrium (LD) independently affected gene expression and phenotype). For sensitivity analyses, a two-sample MR analysis was performed using random-effects inverse-variance weighted, weighted median, simple mode, weight mode and MR-Egger methods [38].

### Sensitivity analysis

Additional sensitivity analyses, such as horizontal pleiotropy and colocalisation analysis, were performed for significant MR associations. Analyses using additional summary statistics were also performed to validate the main outcomes.

#### Colocalisation analysis

For statistically significant MR associations, Bayesian colonisation analyses were performed to evaluate the posterior probability of the same causal variants; the genetic variants with statistically significant MR associations for gene expression and outcomes were assessed in the coloc (v3.1) R package [39]. Default parameters were used for analysis.

#### Horizontal pleiotropy analysis

One genetic variant may be associated with the expression of more than one gene, which could thus violate the MR assumption that the instrumental variable only has an association with an outcome via drug target gene expression changes. We extracted associations with nearby genes (within a 2 Mb window) to evaluate horizontal pleiotropy. SMR analyses were used to test if the expression of these nearby genes was associated with nominal significance (*p* < 0.05), and associated or not, with the outcome. We reported genes that passed nominal significance as we were interested in identifying specific genes which were related to the trait rather than all genes or the study as a whole.

#### MR analysis of gene expression in the brain and MDD

We performed SMR analyses on gene expression in brain tissues and MDD using the PsychENCODE resource, with a sample size of 1387 unique donors. The effects estimate from SMR represented the effect on the risk (OR) of disease per 1-SD change in gene expression. We also performed SMR analysis on gene expression data in the GTEx (v8) dataset at 13 different brain regions, with sample sizes ranging between 130 and 250 unique donors. All association analyses were performed within a 2 Mb window around each gene or transcription start site.

#### Target gene expression profiles in tissue

To analyse the expression of genes and DNAm profiles, we used two whole blood sample datasets (GSE98793 and GSE125105 datasets) downloaded from the Gene Expression Omnibus website (https://www.ncbi.nlm.nih.gov/gds). The GSE98793 dataset used an Affymetrix Human Genome Array which included 64 MDD patients and 64 healthy control whole blood samples. Expression parameters comprised fragmented per kilobase of transcript per million mapped reads with a log2 transformation, which were transformed to transcripts per kilobase million. Moreover, genes with a zero SD in expression were removed (they were irrelevant to further analysis). The GSE125105 dataset used Illumina Infinium 450k Human DNAm bead-chip technology and included 489 MDD patients and 210 healthy controls. Before analyses, datasets were normalised using *z*-scores. *t*-tests were used to identify differences in gene expression levels between MDD patients and healthy controls.

To explore the biological mechanisms of significant genes using analytical strategies, the GTEx Portal (https://gtexportal.org/home/) was used to identify target gene expression levels in 13 brain regions and whole blood samples. We also explored exon expression levels in 54 tissues (GTEx V7).

### MR power calculations

We performed power calculations using an online tool [40] (http://sb452.shinyapps.io/power/) to estimate causal effects at 80% power detection.

### Weak instrumental bias in MR analyses

The *F* value from exposure regression (DNAm or gene expression) in instrumental variables (eQTL or mQTL SNP) was used in one-sample MR studies to assess instrumental variable strength. As a general rule, instrumental variables with an *F* value > 10 were not considered ‘weak instruments’ [41]. For two-sample MR analysis, the *F* value was generated using the approximation report by Bowden et al. [42] (information is provided in Supplementary Methods).

## Results

### Genetic instrument selection

**Fig. 1:**
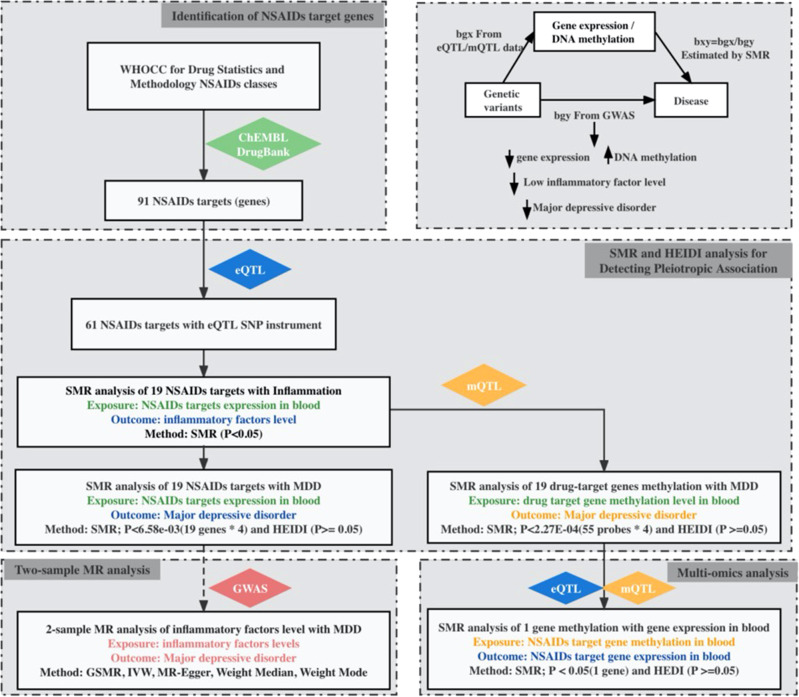
Summary of study design and results. bxy estimated effect of exposure on outcome; eQTL expression quantitative trait loci, mQTL methylation quantitative trait loci, GWAS genome-wide association study, HEIDI heterogeneity in dependent instrument, MR Mendelian randomisation, SMR summary-based MR, SNP single-nucleotide variant, and WHOCC World Health Organization Collaborating Centre, MDD major depressive disorder.

A study design summary and primary results are shown (Fig. 1). In total, 91 gene encoding proteins were identified, which were experimentally shown to be modified by one or more NSAIDs (eTable 3, Supplementary Tables). Of these, 61 genes were expressed in blood as annotated by eQTLGen. Of these, the expression of 19 genes was associated with inflammatory factor levels at *P_SMR* < 0.05 (eTable 4; Supplementary Tables) and these were included in the main analysis. For selected genes, independent *cis*-eQTL SNPs were included that reached GWAS significance (*p* < 5 × 10^−8^). Of these, the most significant *cis*-eQTL SNPs were selected as genetic instruments, with *F* values > 10 (eTable 4, Supplementary Tables).

### MR analysis of gene expression in blood and MDD

**Fig. 2:**
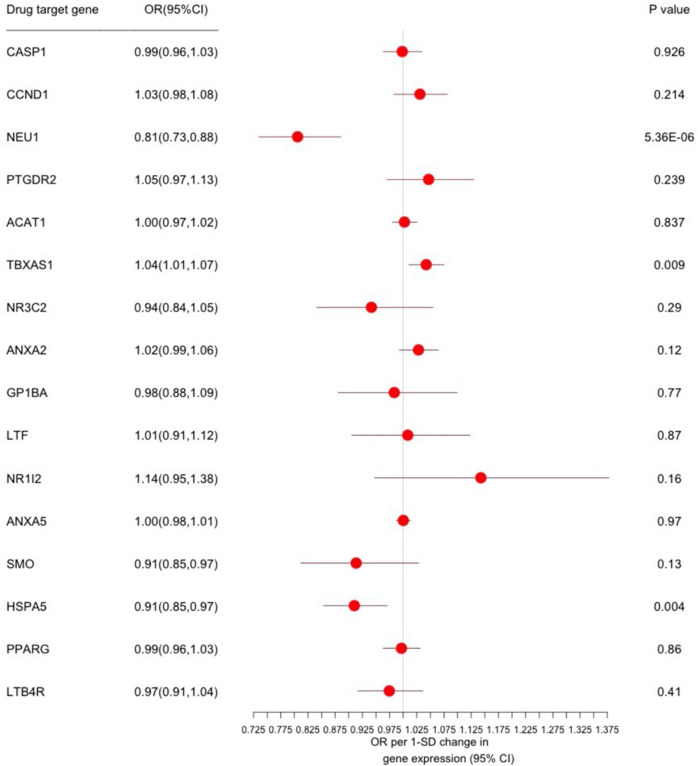
Association drug target gene expression in blood with major depressive disorder (MDD) risk. Forest plot of the association between a 1-SD change in expression of 16 inflammatory factor levels lowering drug target genes in blood with risk for MDD. Data are represented as odds ratios (ORs) with 95% CI (error bars). The direction of gene expression change reflects the inflammatory factor levels lowering association. Therefore, an OR of greater than 1.00 suggests an decreased risk of MDD associated with NSAIDs treatment. Associations are statistically significant after correcting for multiple testing (16 genes × 3 inflammatory factors) and have a heterogeneity in dependent instrument (HEIDI) *p* ≥ 0.05, statistically significant after correcting for multiple testing but have a HEIDI *p* < 0.05 (indicating association likely due to linkage) or did not pass the multiple testing correction. SMR indicates summary-based Mendelian randomisation.

Using a significant threshold of *p* < 6.58 × 10^−4^ and a HEIDI test *p* ≥ 0.05 value to exclude linkage associations, we identified a 1-SD decrease in blood for neuraminidase 1 (*NEU1*) expression, which was associated with decreased CRP levels of 0.277 mg/l (95% CI: 0.128–0.426) (eTable 4, Supplementary Tables) and a lower risk of MDD (OR = 0.806; 95% CI: 0.735–0.885; *p* = 5.36 × 10^−6^) (Fig. 2 and eTable 5, Supplementary Tables). The genetic mutations of top associated SNP (rs367364) regulated gene expression probes (ENSG00000204386) to change the degree of the *NEU1* expression. Decreased *NEU1* expression could lead to decreased CRP levels and a lower risk of MDD. Colocalisation analysis identified a > 85% posterior probability for the same variant (rs367364) regulating *NEU1* (OMIM 608272) expression and affecting MDD risk (eTable 6, Supplementary Tables).

Using CAGE eQTL and MDD (UKB) GWAS datasets, these results indicated a concordant association between *NEU1* expression and MDD risk. The results also indicated that a 1-SD decrease in blood *NEU1* expression was associated with decreased inflammatory factor levels (CRP) of 0.168 mg/l (95% CI: 0.045–0.290) (eTable 7, Supplementary Tables) and a lower risk of MDD-UKB (OR = 0.966; 95% CI: 0.948–0.985, *p* = 5.19 × 10^−4^) (eFig. 1 and eTable 8, Supplementary Tables). From primary analysis, the top SNP (rs3130063) in ENSG00000204386 validation analysis was 46 kilobase pairs (kbp) distant from the top associated SNP (rs367364). The *r*^2^ and *D’* values for rs3130063 and rs367364 were 0.25 and 0.55, respectively.

### MR analysis of DNAm in MDD

We used a significant threshold of *p* < 3.54 × 10^−5^ and a HEIDI *p* ≥ 0.05 value to exclude linkage associations and found that a 1-SD increase in blood *NEU1* DNAm was associated with a lower risk of MDD (OR = 0.886; 95% CI: 0.836–0.939; *p* = 4.71 × 10^−5^) (eTable 9, Supplementary Tables). The genetic mutations of top associated SNP (rs693906) change the degree of *NEU1* DNAm by regulating DNAm probes (cg00397479). Decreased *NEU1* expression could lead to decreased CRP levels and a lower risk of MDD. We observed a reverse association between *NEU1* DNAm and MDD risk using validated mQTL and GWAS datasets (OR = 0.969; 95% CI: 0.954–0.986; *p* = 3.01 × 10^−4^) (eTable 10, Supplementary Tables). The top SNP (rs3130063) in the validation analysis of cg00397479 was 31 kbp distant from the top associated SNP (rs693906) in primary analysis.

### MR associations across multi-omics platforms

**Fig. 3:**
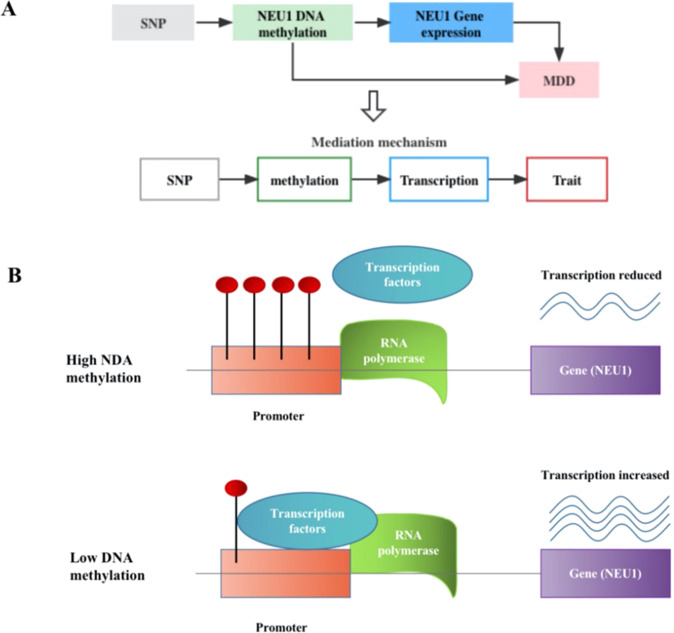
Flowchart to identify mediation mechanism. **A** The effects of DNA methylation (DNAm) on trait, DNAm on gene expression, and gene expression on trait are evaluated using the SMR and HEIDI method and integrated to identify potential mediation mechanisms in which an SNP exerts an effect on the trait by altering the DNAm level, which then regulates the expression levels of a functional gene. The detailed steps were (1) Use SMR to determine association between DNAm and gene expression; (2) Use SMR to determine associations between DNAm and major depressive disorder (MDD); (3) Use SMR to determine associations between gene expression and MDD. **B** When DNAm in the promoter is higher, the binding of transcription factor is disrupted, thereby suppressing the expression of *NEU1*. When DNAm is low, transcription factors usually bind to the promoter, and the expression level of *NEU1* increase.

We integrated two omics results to identify *NEU1* pleiotropy between DNAm and MDD, and between gene expression and MDD. Then, possible associations were explored between DNAm and gene expression using DNAm as the exposure and the transcript as the outcome. The results for these pairs are shown in the median model (Fig. 3A) and indicated that the effects of genetic variants in the MDD regulating the expression of gene mediates loci through DNAm.

**Fig. 4:**
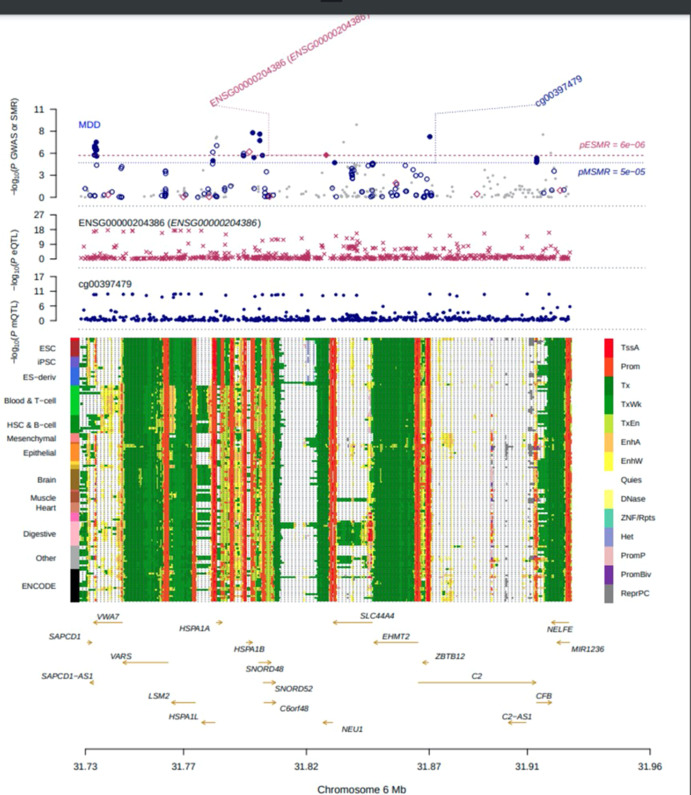
Results of SNP and SMR association across mQTL, eQTL and GWAS at the *NEU1* locus. The top plot shows −log10 (*p* values) of SNPs from the major depressive disorder (MDD) GWAS. The red diamond and blue circle represent −log10 (*p* values) for probes from the SMR tests for associations of gene expression and DNA methylation (DNAm) probes, respectively. The second plot shows eQTL results for the probe ENSG00000204386 (tagging *NEU1*). The third plot shows mQTL results for the DNAm probe cg00397479. The bottom plot shows 18 chromatins (indicated by colours) of 833 samples from EpiMap for different primary cells and tissue types (rows).

Most DNAm sites in DNAm-transcript associations are located in promoter or enhancer regions, making it possible to infer the regulation the degree of DNAm and gene expression by genetic variant. A notable example was the cg00397479-*NEU1*-MDD axis (Fig. 4). *β* values for the causal association between DNAm to MDD, DNAm to gene and gene to MDD were −0.120, −0.519 and 0.215, respectively (eTable 11, Supplementary Tables). Indeed, the DNAm probe (cg00397479) resided in the transcriptional start sites (TSS) region of *NEU1* (ENSG00000204386) across multiple tissues and cells, as identified in the Epigenome Integration Across Multiple Annotation Project (EpiMap) database. The corresponding DNAm locus belonged to the TSS functional region, suggesting a possible biological pathway for disease risk regulation. The genetic mutation (rs693906) regulated the methylation probe (cg00397479) to alter DNAm levels. When these levels at the *NEU1* TSS were high, transcription factor binding was disrupted and suppressed *NEU1* expression (*β* = −0.519; 95% CI: −0.717 to −0.320256; *p* = 3.16 × 10^−7^) and decreased MDD risk (*β* = −0.120; 95% CI: 0.062 to −0.178; *p* = 4.71 × 10^−5^). Furthermore, the top SNP in mQTL and GWAS analysis of the cg00397479 probe was 180 kbp distant from the top associated SNP in the eQTL and GWAS analysis of ENSG00000204386. The *r*^2^ and *D’* values for rs693906 and rs367364 were 0.35 and 0.65, respectively.

From this evidence, we hypothesised that a genetic variant in the TSS functional region of *NEU1* led to DNAm, then down-regulated *NEU1* expression (Fig. 3B). Decreased *NEU1* expression could theoretically reduce MDD risks.

From validation analysis, a concordant association was observed between *NEU1* DNAm, gene expression and MDD risk (eTable 12, Supplementary Tables). *β* values for DNAm to MDD, DNAm to gene and gene to MDD were −0.031, −0.841 and 0.033, respectively. The genetic mutation (rs3130063) regulated the methylation probe (cg00397479) to alter the degree of DNAm. The top SNP (rs3130063) in mQTL and GWAS was the same as the top SNP in eQTL and GWAS analysis of ENSG00000204386.

### Sensitivity analyses

#### MR analyses of *NEU1* brain expression

We failed to observe that *NEU1* expression in all 14 brain regions was associated with MDD risk (eTable 13, Supplementary Tables).

#### Assessing the NEU1 eQTL for horizontal pleiotropy

The eQTL SNP for *NEU1* expression in blood (rs367364) was related to the expression of 42 adjacent genes (*p* < 0.05), including three genes at *p* < 1 × 10^−3^ (*APOM* [OMIM 606907], *TNXB* [OMIM 600985] and *HSPA1A* [OMIM 140550] (eTable 6, Supplementary Tables). However, using SMR and colocalisation analyses, we failed to identify associations between the expression of these adjacent genes with MDD (eTable 14, Supplementary Tables), and we failed to identify horizontal pleiotropy. Possible reasons for the observed associations between *NEU1* expression and MDD are shown (eFig. 2, Supplementary Figs.).

#### MR analysis of inflammatory factor levels in MDD

We identified no evidence to support an association between genetically estimated CRP (OR = 0.1.01, 95% CI: 0.987–1.033; *p* = 0.40), IL-6 (OR = 0.97, 95% CI: 0.942–1.008; *p* = 0.14) and TNF (OR = 1.01, 95% CI: 0.987–1.027; *p* = 0.48) levels in MDD using 56, 9 and 5 SNPs, which were respectively associated with GWAS significance (*p* = 5 × 10^−8^) as MR instruments (after HEIDI outlier filtering to remove pleiotropic SNPs) (eTable 15, Supplementary Tables).

#### NEU1 expression and DNAm profiles and tissue enrichment analysis

We integrated *NEU1* expression and DNAm profile*s* from GSE98793 and GSE125105 datasets and observed no differences in profiles between MDD patients and healthy controls (eFig. 3, Supplementary Figs.).

The eQTL SNP (rs367364) affected *NEU1* expression by affecting of transcription regulation combinations. The T allele (eFig. 4A, Supplementary Figs.) was associated with lower gene expression (*p* = 2.12 × 10^−3^). Assuming that differences in gene expression were related to differences in protein activity, patients carrying a T allele at this eQTL SNP (genotype TT) of *NEU1* were similar to the treatment group of the NSAIDs .

Using GTEx project data, *NEU1* expression was higher in cerebellar and cerebellum regions when compared with other brain regions (eFig. 4B, Supplementary Figs.). Also, exon 5 of *NEU1* was highly expressed in adrenal gland tissue (eFig. 4C, Supplementary Figs.).

## Discussion

Using QTL and GWAS summary statistics, we used a two-sample MR approach to infer the potential effects of NSAIDs towards MDD. Lower *NEU1* expression and higher DNAm *NEU1* expression levels were associated with decreased inflammatory factor (CRP) levels and decreased MDD risk. Our integrative analyses, based on multi-omics datasets, showed that higher DNAm levels were associated with lower *NEU1* expression. Combined results indicated that NSAIDs targeting gene expression or DNAm could decrease MDD risk. We did not observe any association between genetic inflammatory factor level estimates and MDD. Our results indicated that even minor potential effects of inflammatory factor levels (CRP, IL-6, TNF and IL-1) on MDD are not likely and reveal that any association between *NEU1* and MDD is independent of inflammatory factors (CRP, IL-6, TNF and IL-1).

*NEU1* is one of four mammalian neuraminidase isoenzymes and is predominately located in the lysosome [43, 44]. The protein is encoded by the sialidase gene (*NEU1*) and plays a crucial role in the lysosomal catabolism of sialylated glycoconjugates [45]. In addition to this crucial role, growing evidence now suggests that *NEU1* is vital for the immune system and is mechanistically involved in cell signalling during immune responses [44–46]. Recently, it was shown that *NEU1* regulated toll-like receptor (TLR) activation on macrophages; specifically, ligand binding to TLRs induced *NEU1* activity and led to receptor activation, nitric oxide and proinflammatory cytokine production [47]. Importantly, these *NEU1* roles in macrophages are confirmed by several in vitro studies [47–51].

Animal studies [14, 52, 53] also confirmed that *NEU1* deficiency diminished lymphocyte and macrophage stimulation, thereby decreasing immune cell availability for cytokine and antibody production [54]. Critically, global or macrophage-specific *NEU1* knockout mice were associated with improved vascular inflammation, lowered apoptosis, decreased reactive oxygen species production, mitigated extracellular matrix degradation and improved M2 macrophage polarisation [52]. As *NEU1* appears to be required for inflammatory signalling in microglia, the gene could function as a novel target to reduce central nervous system inflammation-related neurodysfunction disorders, including MDD [5, 48]. Abnormal activation of microglia, which are immunological guardians of the brain, and increased microglial cell numbers were observed in depression and anxiety disorders, although it was unclear how this related to psychopathological conditions [55]. Recently, a study described depression as a microglia-associated disorder, and apart from excessive microglia activation and increased cell numbers, microglial decline and senescence were observed in patients with depression [56]. Therefore, microglial activation may suppress neurogenesis and neuroplasticity, further promoting the incidence of depression-like symptoms [57]. From our conclusions, we speculate that *NEU1* could participate in MDD pathogenesis by regulating microglia or macrophages, which in turn regulate cytokine levels, glutamate secretion and HPA axis activity. However, how *NEU1* directly affects MDD pathogenesis remains unknown as limited studies have focused on *NEU1* activities.

Ikeda et al. recently conducted a behavioural analysis using *NEU1*-knockout (*NEU1*-KO) zebrafish [58] and reported that fish showed regular swimming capabilities similar to wild-types but they exhibited lower social interactions, aggression and abnormal social preferences [58], indicating a potential effect of *NEU1* on emotional activities. Notwithstanding this, the extent to which brain function is conserved between fish and mammals is controversial; the distribution and structure of sialoglycans and associated enzymes differ between fish and humans [58]. Thus, elucidating abnormal behaviour and emotional reaction mechanisms in *NEU1*-KO mammalian models and cell lines is clinically warranted. Herein, our results indicated that NSAIDs decreased *NEU1* expression or increased *NEU1* DNAm which could reduce MDD risk. Given this evidence, we hypothesise that decreased *NEU1* may act in a negative feedback loop to reduce inflammation by monocytes/macrophages and potentially relieve MDD development.

Although, our study indicated a protective effect of *NEU1*, the use of acetylsalicylic acid to target MDD and its impact on MDD remains controversial in population-based studies [59–61]. Recently, an RCT involving 19,114 older adults showed that taking low-dose aspirin (acetylsalicylic acid) at 100 mg/day did not lower depression rates when compared with placebo [59]. Moreover, a meta-analysis of 12 observational, 5 case-control and 7 prospective cohort studies showed a significant association between aspirin use and depression (OR/Risk ratio = 1.10; 95% CI: 1.05–1.16) [60]. Another systematic review of six clinical studies indicated that the low-dose aspirin (80–100 mg/day) was safe, well-tolerated and potentially efficacious for improving depressive symptoms in patients with unipolar and bipolar depression [61]. These studies indicated that other important information, including drug dose and exposure duration, inter-individual variation with respect to drug metabolism and genetics exerted modifying roles in terms of drug efficacy and toxicity. Thus, conducting specific RCTs for different dose subgroups and drug exposure durations could help characterise the cumulative exposure effects on outcomes. Performing MR analysis does have a well-defined interpretation of causal relationship as an intervention on the genetic code occurs at conception. Thus, MR estimates could represent a life-long difference in exposure between genetic subgroups [62]. Furthermore, most RCTs are performed on mature individuals, who may in a stage of disease progression is irreversible. There may be no intervention on the exposure in a mature cohort which can imitate the genetic effect [62]. Hence, causation assessments using MR investigations and clinical interventions could identify true causal exposure associations for MDD and provide more evidence for MDD prevention.

No causal association between inflammatory factor levels and MDD was identified by using MR method, our results suggested that any associations between *NEU1* and MDD were not likely to be mediated by the changes of inflammatory factors (CRP, IL-6 and TNF) levels. Other two-sample MR analyses focused on causal relationships between cytokines; MDD did not consistently demonstrate CRP or IL-6 associations with other depressive symptoms, except for an association between IL-6 signalling and suicidality [21]. The association between inflammation and MDD is unequivocal; over the past two decades, growing evidence has suggested that MDD is associated with systemic immune activation, comprising abnormalities in inflammatory makers, immune cell numbers and antibody titres [5]. Our results do not show a deniable association between immune system activity and MDD, thus, it is plausible that other inflammatory profiles, besides CRP, IL-6 and TNF, may be associated with different depression subtypes [63, 64]. Conducting more comprehensive and high-quality studies could facilitate a better understanding of immune-neuropsychological interactions. By further exploring the relationships arising between immunity and MDD, we can improve our understanding of disease conditions and help design better therapies to promote the quality of life of individuals with MDD.

Besides NSAIDs, we also identified *NEU1* as a target for oseltamivir. Oseltamivir was developed for prophylactic and therapeutic use against influenza, specifically targeting the viral enzyme’s highly-conserved active site [65]. It has been reported that some oseltamivir-treated children had experienced adverse neuropsychiatric events [66–68]. To estimate the incidence rate of adverse neuropsychiatric events in patients given oseltamivir, Toovey et al. conducted an analysis covering Japan, the US, and other countries and finding such neuropsychiatric events generally fell into the categories of abnormal behaviours, delusions, and perceptual disturbances [66]. In addition, there were cases of delirium and delirium-like events, depressed consciousness levels, parasomnia, suicidal events, accidents, and injuries [66]. One 15-year-old girl in Korea was diagnosed with depressive episode after taking oseltamivir [68]. Reportedly, the neuropsychiatric adverse events were more common in children than in adults [67]. Based on the neuropsychiatric reverse effect of oseltamivir in children, the implication of *NEU1* targeted drugs, including NSAIDs and oseltamivir, for the effect of treatment for depression should be explained with caution in both children and teenagers.

## Study limitations and strengths

Firstly, our assumption that alterations in gene expression exerted changes in protein levels and activity may not always be the case. Therefore, our failure to detect associations may not indicate a lack of biological effects of drug therapy. Secondly, our study did not detect associations between expression changes in drug target genes and DNAm in specific tissues with MDD. For example, we did not detect significant associations between *NEU1* and MDD in brain tissue, which may have been restricted by eQTL dataset sample size. Given this and varying instrument strength in different brain regions (eTable 13), our study did not identify any smaller but clinically relevant effects of *NEU1* expression in any brain regions. Moreover, a lack of statistical power in identifying outcomes could also be another viable explanation for some of our study limitations (for power calculations, eTable 16 Supplementary Tables). Thirdly, we did not estimate any associations between NSAIDs and other inflammatory factors or MDD sub-symptoms—factors worth exploring in the future. Lastly, estimates from our MR analyses may not have reflected the actual effects of drug exposure. Other important information, such as doses and drug exposure duration, individual drug metabolism variants, the ability to reach target tissue and drug-binding affinity could have exerted modifying effects on drug efficacy and toxicity, thus making it difficult to extrapolate the effects between drug exposure and our analyses.

However, our study had some unique strengths. Human genetics continuously provides evidence for new drug discovery and drug safety. Loss-of-function mutations in drug target genes are ideal MR instrumental variables, however, as these variants are rare, related MR studies are largely unfeasible. Alternatively, we used eQTL and mQTL genetic variants as MR instrumental variables. Available GWAS, eQTL and mQTL datasets were exploited to investigate causality, and we avoided observational study and RCT limitations by using a two-sample MR design, e.g., limited sample size and reduced confounding bias, while using *cis*-SNPs to represent messenger RNA or DNAm levels could help to minimise the potential for horizontal pleiotropy. We also explored associations between DNAm and gene expression, which could help unravel biological mechanisms underpinning inflammation in MDD.

## Conclusions

We identified a concordant association for lower *NEU1* messenger RNA and higher DNAm levels with MDD risk, with sensitivity analyses suggesting that any *NEU1* association with MDD were likely to be independent of inflammatory factor associations. We speculate that the further lowering of *NEU1* activity or increasing *NEU1* DNAm expression in patients with MDD could improve symptoms or limit disease episodes. Our findings warrant further investigations into the functional role of *NEU1* in MDD and the potential exploitation of drug-repurposing for this condition.

## Supplementary information

Supplementary tables

Supplementary figures

Supplementary methods

## Data Availability

All the data used in this study could be accessed online. The links for data used in this study can be found in the Mendeley data (10.17632/xj67ctfpkw.1).
