# Supplementary material for: Non-steroidal anti-inflammatory drug target gene associations with major depressive disorders: a Mendelian randomisation study integrating GWAS, eQTL and mQTL Data
Source: Pharmacogenomics J. 2023 Mar 25;23(4):95–104. doi: 10.1038/s41397-023-00302-1 (PMC10382318; doi:10.1038/s41397-023-00302-1)
Supplement: Supplementary file 1 — Supplementary tables [file 41397_2023_302_MOESM1_ESM.pdf]

Index	Title
eTable 1	Expression(DNA methylation) quantitative trait loci (eQTL/mQTL) datasets
eTable 2	Genome-wide association studies used in Mendelian randomisation analysis
eTable 3	Target genes for Non-Steroidal Anti-Inflammatory Drugs (NSAIDs) identified using the DrugBank and ChEMBL databases
eTable 4	MR association between drug target gene expression in blood and inflammatory factor levels(Psmr_threshold<0.05)
eTable 5	MR association between drug target gene expression in blood and major depressive disorder (Psmr_threshold < 0.00104[0.05/16/3])
eTable 6	MR association between expression in blood of genes adjactent to NEU1 and MDD risk(Psmr_threshold < 0.00116[0.05/43])
eTable 7	Validated MR association between drug target gene expression in blood and inflammatory factor levels (Psmr_threshold < 0.05)
eTable 8	Validation of MR association between drug target gene expression in blood and MDD by using UK Biobank database(Psmr_threshold < 0.0011[0.05/15/3])
eTable 9	MR association between drug target gene DNA methylation in blood and MDD(Psmr_threshold < 0.00035461[0.05/47/3])
eTable 10	Validation MR association between drug target gene DNA methylation in blood and MDD (UKB)(Psmr_threshold < 0.0007936507[0.05/21/3])
eTable 11	eTable 11. MR association between NEU1 DNA methylation in blood and gene expression (Psmr_threshold < 0.05 & PHEIDI_threshold > 0.05)
eTable 12	Validation of MR association analysis, gene NEU1 DNA methylation in blood and gene expression
eTable 13	Association Between NEU1 Brain Expression and MDD Risk(Psmr_threshold < 0.0035[0.05/14])
eTable 14	Association between NEU1 eQTL SNP (rs367364) in blood with expression of other nearby genes (P_threshold < 0.05)
eTable 15	Causal estimates from different MR methods for the association of inflammantory factors level with Major depression disorder
eTable 16	Association Between NEU1 Blood Gene Expression and MDD risk(Psmr_threshold < 0.00333[0.05/16])

eTable 1. Expression(DNA methylation) quantitative trait loci (eQTL/mQTL) datasets

Tissue	Dataset	Sample size	Sample Ancetsry	Reference	Data download
Blood	eQTLGen Consortium	31,684 (meta-analysis of 37 studies)	Predominantly European (~95%)	https://www.biorxiv.org/content/10.1101/447367v1	Original data: https://www.eqtlgen.org/cis-eqtls.html; SMR Format Data: https://cnsgenomics.com/software/smr/#DataResource
Blood	CAGE eQTL	2,765	European ancestry	https://www.cell.com/ajhg/fulltext/S0002-9297(16)30532-8	
Blood	LBC_BSGS mQTL	1,980	European descent	https://www.nature.com/articles/s41467-018-03371-0	
Blood	Hannon mQTL	1,175	European descent	https://www.sciencedirect.com/science/article/pii/S0002929718303185?via=ihub	
Brain - Cerebellum	GTExV8	241	Predominantly European (~85%) , ~67% male	https://www.ncbi.nlm.nih.gov/pubmed/29022597	Original data: https://www.gtexportal.org/home/datasets; SMR Format Data: https://cnsgenomics.com/software/smr/#DataResource
Brain - Cortex		255			
Brain - Nucleus accumbens (basal ganglia)		246			
Brain - Caudate (basal ganglia)		246			
Brain - Cerebellar Hemisphere		215			
Brain - Frontal Cortex (BA9)		209			
Brain - Hypothalamus		202			

Brain - Putamen (basal ganglia)		205			
Brain - Hippocampus		197			
Brain - Anterior cingulate cortex (BA24)		176			
Brain - Amygdala		152			
Brain - Spinal cord (cervical c-1)		159			
Brain - Substantia nigra		139			
Brain - Prefrontal Cortex	PsychENCODE	1387 (meta-analysis of 35 eQTL studies, including data from GTEx)	Predominantly European, ~65% male	https://www.ncbi.nlm.nih.gov/pubmed/30545857	Original data: http://resource.psychencode.org/; SMR Format Data: https://cnsgenomics.com/software/smr/#DataResource

eTable 2. Genome-wide association studies used in Mendelian randomisation analysis

Disease	Number of cases	Number of controls	Ancestry	PMID	Authors	Publication date	Data download
C-reactive protein level	204,402		European	30388399	Ligthart et al.	2018	ieu open gwas project
interleukin-6 measurement	21,758		European	33067605	Folkersen L et al.	2020	GWAS catalog
TNF-related apoptosis-inducing ligand measurement	1,301		European	33303764	Gilly A et al.	2020	GWAS catalog
interleukin-1 beta measurement	4,910		European	30206230	Offenbacher S et al.	2018	GWAS catalog
Major depressive disorder	170,756	329,443	European	29700475	Wary NR et al.	2018	PGC consotium (exclude UKB and 23andME
Major depressive disorder/Broad depression	113,769	208,811	European	29662059	Howard et al.	2018	GWAS ALATS

eTable3. Target genes for Non-Steroidal Anti-Inflammatory Drugs (NSAIDs) identified using the DrugBank and ChEMBL databases

Drug subclass	ATC code	Medication Subclass	ATC code	Drug name	ATC code	Target genes	Drug description
TOPICAL PRODUCTS FOR JOINT AND MUSCULAR PAIN	M02A	Antiinflammatory preparations, non-steroids for topical use	M02AA	Aceclofenac	M02AA25	PTGS2/PTGS1	Aceclofenac is indicated for the relief of pain and inflammation in osteoarthritis, rheumatoid arthritis and ankylosing spondylitis.
				Benzydamine	M02AA05		A locally-acting NSAID indicated for the symptomatic relief of pain in acute sore throat and for the symptomatic relief of oropharyngeal mucositis caused by radiation therapy.
				Bufexamac	M02AA09	PTGS1/PTGS2/HDAC6/HDAC10	An NSAID used to treat skin conditions like atopic eczema and inflammatory dermatoses.
				Suxibuzone	M02AA22	NA	Not Annotated
				Etofenamate	M02AA06	NA	An NSAID used to treat muscle and joint pain.
				Felbinac	M02AA08	CTSL	Not Available
				Nifenazone	M02AA24	NA	Not Annotated

				Niflumic acid	M02AA17	PLA2G1B/PTGS2/CLCNKA/PTGS1/PLA2G4A/UGT1A9	A cyclooxygenase-2 inhibitor used to alleviate inflammation, pain, and edema associated with acute and chronic inflammatory conditions, such as rheumatoid arthritis, osteoarthritis, post-operative inflammatory conditions, and physical trauma.
				Nimesulide	M02AA26	PTGS2/PLA2G2E/LTF	A cyclooxygenase 2 inhibitor used to treat acute pain and primary dysmenorrhea.
				Fentiazac	M02AA14		Not Annotated
ANTIINFLAMMATORY AND ANTIRHEUMATIC PRODUCTS, NON-STEROIDS	M01A	Propionic acid derivatives	M01AE	Dexketoprofen	M01AE17	PTGS1/PTGS2	An NSAID that is the R(-)-enantiomer of racemic ketoprofen with analgesic and anti-inflammatory properties used for the treatment of mild to moderate pain.
				Flurbiprofen	M01AE09	PTGS1/PTGS2	An NSAID used to treat the signs and symptoms of osteoarthritis and rheumatoid arthritis.

				Naproxen	M01AE02	PTGS1/PTGS2/PAB	An NSAID used to treat rheumatoid arthritis, osteoarthritis, ankylosing spondylitis, polyarticular juvenile idiopathic arthritis, tendinitis, bursitis, acute gout, primary dysmenorrhea, and mild to moderate pain.
				Ketoprofen	M01AE03	CXCR1/PTGS1/PTGS2	An NSAID used to treat rheumatoid arthritis, osteoarthritis, ankylosing spondylitis, dysmenorrhea, mild to moderate muscle pain, postoperative pain, and postpartum pain.
				Ibuprofen	M01AE01	BCL2/THBD/FABP2/PPARG/CFTR/PPARA/GP1BA/S100A7	An NSAID and non-selective COX inhibitor used to treat mild-moderate pain, fever, and inflammation.

				Alminoprofen	M01AE16		Alminoprofen is a non-steroidal anti-inflammatory drug (NSAID) whose physiochemical characteristics make it a member of the phenylpropionic acid class of chemical substances.
				Dexibuprofen	M01AE14	PTGS2/PTGS1/BCL2/THBD/PLAT/FABP2/PPARG/CFTR/SLC15A1/PPARA/GP1BA/S100A7	A pharmacologically active enantiomer of racemic ibuprofen (NSAID) used to treat pain and inflammation.
				Benoxaprofen	M01AE06		The use of benoxaprofen, formerly marketed as Oraflex tablets, was associated with fatal cholestatic jaundice among other serious adverse reactions. The holder of the approved application voluntarily withdrew Oraflex tablets...

				Fenbufen	M01AE05	NA	Fenbufen is a non-steroidal anti-inflammatory drug used primarily to treat inflammation in osteoarthritis, ankylosing spondylitis, and tendinitis. It can also be used to relieve backaches, sprains, and fractures. Fenbufen is...
				Pirprofen	M01AE08		Not Annotated
				Ibuproxam	M01AE13		Ibuproxam is a non steroidal anti-inflammatory drug (NSAID).
				Fenoprofen	M01AE04	PPARA/PPARG	An anti-inflammatory analgesic used to treat mild to moderate pain in addition to the signs and symptoms of rheumatoid arthritis and osteoarthritis.
				Oxaprozin	M01AE12	PTGS1/PTGS2	An NSAID used to treat osteoarthritis, rheumatoid arthritis, and juvenile rheumatoid arthritis.

				Indoprofen	M01AE10		A drug that has analgesic and anti-inflammatory properties. Following reports of adverse reactions including reports of carcinogenicity in animal studies it was withdrawn from the market worldwide.
				Suprofen	M01AE07	PTGS1/PTGS2	An NSAID used to prevent pupil constriction in ocular surgery.
				Tiaprofenic acid	M01AE11	PTGS1/PTGS2	A nonsteroidal anti-inflammatory (NSAID) used to manage inflammation and analgesia associated with rheumatoid arthritis and osteoarthritis.
				Flunoxaprofen	M01AE15		Not Annotated
		Other antiinflammatory and antirheumatic agents, non-steroids	M01AX	Feprazone	M01AX18		Not Annotated
				Tenidap	M01AX23	PTGS1/PTGS2	Not Annotated
				Azapropazone	M01AX04	NA	Not Available
		Butylpyrazolidines	M01AA	Mofebutazone	M01AA02		Not Annotated

				Phenylbutazone	M01AA01	PTGIS	An NSAID used to treat backache and ankylosing spondylitis.
				Oxyphenbutazone	M01AA03	PLA2G2E	A nonsteroidal anti-inflammatory drug (NSAID) no longer commonly used for the symptomatic relief of musculoskeletal pain.
		Acetic acid derivatives and related substances	M01AB	Tolmetin	M01AB03	PTGS1/PTGS2	An NSAID used to treat acute flares of various painful conditions and used for the long term management of osteoarthritis, rheumatoid arthritis, and juvenile arthritis.
				Acemetacin	M01AB11	PTGS1/PTGS2	A NSAID indicated in the treatment of pain and inflammation.
				Alclofenac	M01AB06	PTGS2	Alclofenac is indicated in rheumatology, in particular for the treatment of rheumatoid arthritis, ankylosing spondylitis and, as an analgesic, in painful arthritic pathologies.

				Bumadizone	M01AB07		Bumadizone has been approved for use in Germany and Austria, it is a drug with anti-inflammatory, antipyretic, and analgesic properties, and was marketed for the treatment of both rheumatoid arthritis...
				Difenpiramide	M01AB12	NA	Not Annotated
				Etodolac	M01AB08	RXRA/PTGS1/PTGS2	An NSAID used to treat osteoarthritis and rheumatoid arthritis, as well as acute pain.
				Proglumetacin	M01AB14		A non-steroidal anti-inflammatory medication used to manage pain associated with various inflammatory conditions including rheumatoid arthritis and osteoarthritis.
				Sulindac	M01AB02	AKR1B1/MAPK3/PPARD/PTGDR2/AKR1B10	An NSAID used to treat osteoarthritis, rheumatoid arthritis, ankylosing spondylitis, acute subacromial bursitis or supraspinatus tendinitis, and acute gouty arthritis.
				Lonazolac	M01AB09		Not Annotated

				Zomepirac	M01AB04	PTGDR2	Zomepirac was indicated for the management of mild to severe pain.
				Ketorolac	M01AB15		An NSAID used to treat moderate to severe pain, rheumatoid arthritis, osteoarthritis, ankylosing spondylitis, menstrual disorders, and headaches.
				Indomethacin	M01AB01	PLA2G2A/PTGR2/PPARG/GLO1/PTGDR2/PPARA/AKR1C3/EIF2AK2	A nonsteroidal anti-inflammatory (NSAID) used for symptomatic management of chronic musculoskeletal pain conditions and to induce closure of a hemodynamically significant patent ductus arteriosus in premature infants.
		Coxibs/selective COX-2 inhibitors	M01AH	Celecoxib	M01AH01	PDPK1/CA2/CA3/CDH11	An NSAID used to treat osteoarthritis, rheumatoid arthritis, acute pain, menstrual symptoms, and to reduce polyps is familial adenomatous polyposis.

				Valdecoxib	M01AH03	CA2/PTGS1/PTGS2	A COX-2 inhibitor used to treat osteoarthritis and dysmenorrhoea.
				Polmacoxib	M01AH07		Polmacoxib has been used in trials studying the treatment of Osteoarthritis, Osteoarthritis, Hip, Osteoarthritis, Knee, Localized Primary Osteoarthritis of Hip, and Localized Primary Osteoarthritis of Knee.
				Etoricoxib	M01AH05	PTGS2	A selective COX-2 inhibitor used to relieve moderate post-surgical dental pain as a short-term treatment and inflammatory and painful symptoms of various forms of arthritis.
				Parecoxib	M01AH04		A selective COX-2 inhibitor and NSAID used for the short-term management of perioperative pain.

				Rofecoxib	M01AH02	ELN/PTGS2	A COX-2 inhibitor NSAID used to treat osteoarthritis, rheumatoid arthritis, acute pain, primary dysmenorrhea, and migraine attacks.
				Lumiracoxib	M01AH06	PTGS1/PTGS2	For the acute and chronic treatment of the signs and symptoms of osteoarthritis of the knee in adults.
		Other antiinflammatory and antirheumatic agents, non-steroids	M01AX	Proquazone	M01AX13		Not Annotated
				Morniflumate	M01AX22	ALOX5/PTGS2/LTB4R/TBXA2R	An NSAID derived from niflumic acid used in the symptomatic treatment of inflammatory conditions of the airways, ENT, and the urogenital and osteoarticular systems.
				Nabumetone	M01AX01	PTGS1/PTGS2	An NSAID used to treat osteoarthritis and rheumatoid arthritis.
		Oxicams	M01AC	Droxicam	M01AC04	PTGS1/PTGS2	Droxicam is an NSAID previously used for the treatment of inflammation and rheumatoid arthritis .

				Tenoxicam	M01AC02	PTGS1/PTGS2	An anti inflammatory analgesic used to treat mild to moderate pain as well as the signs and symptoms of rheumatoid arthritis and osteoarthritis.
				Lornoxicam	M01AC05	PTGS1/PTGS2	An NSAID indicated in the treatment of mild to moderate pain, as well as rheumatoid arthritis and osteoarthritis.
				Piroxicam	M01AC01	PTGS1/PTGS2	An NSAID used to treat the symptoms of osteoarthritis and rheumatoid arthritis.
		Fenamates	M01AG	Meclofenamic acid	M01AG04	PTGS1/PTGS2	An NSAID used to treat mild to moderate pain, primary dysmenorrhea, heavy menstrual blood loss, rheumatoid arthritis, and osteoarthritis.
				Tolfenamic acid	M01AG02	PTGS1/PTGS2	In the information for tolfenamic acid, it is stated that this drug, being an NSAID, is effective in treating the pain associated with the acute attack of migraines in adults.

				Mefenamic acid		PTGS1/PTGS2	An NSAID used to treat mild to moderate pain for no more than a week, and primary dysmenorrhea.
STOMATOLOGICAL PREPARATIONS	N02B	Salicylic acid and derivatives	N02BA	Acetylsalicylic acid	N02BA51	PTGS1/PTGS2/AKR1C1/EDNRA/TP53/HSPA5/RPS6KA3/NFKBIA/TNFAIP6/CASP1/CASP3/IKBKB/CCND1/MYC/PCNA/NEU1	A salicylate used to treat pain, fever, inflammation, migraines, and reducing the risk of major adverse cardiovascular events.
				Ethenzamide	N02BA07	NA	Not Annotated
				Diflunisal	N02BA11	PTGS1/PTGS2	An NSAID used to treat mild to moderate pain, inflammation, osteoarthritis, and rheumatoid arthritis.
				Guacetisal	N02BA14		Not Annotated
				Benorilate	N02BA10		Not Annotated
				Imidazole salicylate	N02BA16		Not Annotated

				Salicylamide	N02BA71		Salicylamide is the common name for the substance o-hydroxybenzamide, or amide of salicyl. Salicylamide is a non-prescription drug with analgesic and antipyretic properties. It has similar medicinal uses to aspirin....
				Salsalate	N02BA06		A nonsteroidal anti-inflammatory agent used in the symptomatic relief of rheumatoid arthritis, osteoarthritis and related rheumatic disorders.
		Pyrazolones	N02BB	Aminophenazone	N02BB03		An analgesic drug used to treat acute migraine attacks in combination with ergotamine and caffeine.
				Propyphenazone	N02BB04		Not Annotated
				Metamizole	N02BB02	PTGS1	An antipyretic and analgesic drug used to relieve severe and persistent fever and pain.

		Other analgesics and antipyretics	N02BG	Floctafenine	N02BG04		An anti-inflammatory analgesic used to manage mild to moderate acute pain.
		Anilides	N02BE	Propacetamol	N02BE05	TRPV1/CNR1	Propacetamol is a paracetamol prodrug of intravenous administration used to control fever and pain of perioperative period in multimodal analgesia therapy.
ANTITHROMBOTIC AGENTS	B01A	Platelet aggregation inhibitors excl. heparin	B01AC	Carbaspirin calcium	B01AC08		An NSAID indicated in the treatment of short term pain and fever.
				Indobufen	B01AC10		A reversible platelet aggregation inhibitor used to prevent coronary and peripheral artery occlusion.
ANTIINFLAMMATORY AGENTS	S01B	Antiinflammatory agents, non-steroids	S01BC	Nepafenac	S01BC10	PTGS1/PTGS2	An ophthalmic NSAID used for the symptomatic treatment of pain and inflammation associated with cataract surgery.
			S01BC	Bromfenac	S01BC11	PTGS1/PTGS2	An NSAID used to treat postoperative pain and inflammation of the eye.

			S01BC	Salicylic acid	S01BC08		An acid used to treat acne, psoriasis, calluses, corns, keratosis pilaris, and warts.
			S01BC	Pranoprofen	S01BC09		A topical anti-inflammatory agent used for the treatment of non-infectious inflammatory conditions such as blepharitis and conjunctivitis. Pranoprofen ophthalmic solution may also be used post-operatively to manage inflammation.
			S01BC	Bendazac	S01BC07	PTGS1/PTGS2	A non-steroidal anti-inflammatory drug (NSAID) available as an eye drop for the treatment of cataracts and as a topical cream for the treatment of dermatitis, eczema, hives, skin ulcers and other inflammatory skin conditions.
OTHER OTOLOGICALS	S02D	Analgesics and anesthetics	S02DA	Antipyrine	S02DA03 — Phenazone	PTGS1/PTGS2	An antipyretic agent used for the symptomatic treatment of acute otitis media, most commonly in combination with benzocaine.

INTESTINAL ANTIINFLAMMATORY AGENTS	A07E	Aminosalicylic acid and similar agents	A07EC	Balsalazide	A07EC04	PPARG/PTGS2/PTGS1/ALOX5	An aminosalicylate used to treat ulcerative colitis.
				Mesalazine	A07EC02	ALOX5/PPARG/CHUK/IKBKB/MPO	An aminosalicylate drug used to treat mild to moderate active ulcerative colitis and also to maintain remission once achieved.
				Olsalazine	A07EC03	TPMT/IFNG	An anti-inflammatory agent used in the treatment of inflammatory bowel disease and ulcerative colitis.
				Sulfasalazine	A07EC01	ALOX5/PTGS2/PTGS1/PPARG/CHUK/IKBKB/SLC7A11/ACAT1/TBXAS1/PLA2G1B	An anti-inflammatory drug used to treat Crohn's disease and rheumatoid arthritis.
IMMUNOSUPPRESSANTS	L04A	Selective immunosuppressants	L04AA	Apremilast	L04AA32	PDE4	A non-steroidal medication used for the treatment of inflammatory conditions such as psoriasis and psoriatic arthritis.
OTHER DERMATOLOGICAL PREPARATIONS	D11A	Agents for dermatitis, excluding corticosteroids	D11AH	Crisaborole	D11AH06	PDE4A/PDE4B/PDE4C/PDE4D	A non-steroidal topical medication used for the treatment of mild-moderate atopic dermatitis.
ANTIINFLAMMATORY AGENTS	S01B	Anti-Inflammatory Agents, Non-Steroidal	S01BC	Betulinic Acid			Betulinic Acid has been used in trials studying the treatment of Dysplastic Nevus Syndrome.

eTable 4. MR association between drug target gene expression in blood and inflammatory factor levels(Psmr_threshold<0.05)

Probe ID	Gene	Chromos ome	Base pair	top eQTL SNP	SNP Chromosome	SNP base pair	coded allele	other allele	coded allele frequency	eQTL association			
										beta	se	p-value	F-statistic
ENSG00000137752	CASP1	11	104934164	rs1483025	11	105005936	T	G	0.4008	0.0950381	0.00804494	3.33E-32	139.56
ENSG00000110092	CCND1	11	69462548	rs17318844	11	69402981	A	G	0.4202	-0.126831	0.00800872	1.74E-56	250.80
ENSG00000204386	NEU1	6	31828059	rs2242664	6	31839331	C	T	0.4243	-0.0686133	0.0122131	1.93E-08	31.56
ENSG00000183134	PTGDR2	11	60620928	rs530963	11	60617834	C	A	0.4489	0.113773	0.00893556	3.90E-37	162.12
ENSG00000075239	ACAT1	11	108005373	rs4550189	11	107986344	G	A	0.4172	0.377586	0.0116896	6.77E-229	1043.36
ENSG00000059377	TBXAS1	7	139598487	rs3801148	7	139694500	T	C	0.5061	0.154106	0.00792468	3.13E-84	378.16
ENSG00000151623	NR3C2	4	149182881	rs2358182	4	148978406	C	T	0.4325	-0.0733372	0.00813358	1.94E-19	81.30
ENSG00000182718	ANXA2	15	60667207	rs11071531	15	60727843	C	T	0.4305	0.0922878	0.00811436	5.67E-30	129.35
ENSG00000185245	GP1BA	17	4836958	rs67059207	17	4869093	C	T	0.2106	0.0971946	0.0100672	4.70E-22	93.21
ENSG00000012223	LTF	3	46501930	rs4683221	3	46434962	G	A	0.3180	0.0517643	0.010243	4.34E-08	25.54
ENSG00000144852	NR1I2	3	119518331	rs3732357	3	119530858	G	A	0.2679	-0.0516881	0.00879438	4.17E-09	34.54
ENSG00000164111	ANXA5	4	122603689	rs62320625	4	122597070	C	T	0.1697	0.791354	0.00926554	0	7294.58
ENSG00000128602	SMO	7	128841049	rs7798321	7	128804041	G	A	0.1973	-0.0913166	0.0103391	1.03E-18	78.01
ENSG00000044574	HSPA5	9	128000370	rs2416962	9	128118864	G	A	0.0205	0.714436	0.264183	7.45E-09	7.31
ENSG00000132170	PPARG	3	12402361	rs1699346	3	12504295	G	A	0.4294	-0.115859	0.04129	5.31E-10	7.87
ENSG00000213903	LTB4R	14	24783949	rs3181256	14	24800879	G	A	0.3180	-0.096951	0.046843	3.89E-10	4.28
ENSG00000136997	MYC	8	128750677	rs4733812	8	128999640	A	G	0.383436	-0.0798668	0.00908176	1.44E-18	77.34
ENSG00000151012	SLC7A11	4	139124377	rs6850624	4	139184664	C	T	0.123722	0.0979512	0.0114767	1.40E-17	72.84
ENSG00000184588	PDE4B	1	66549228	rs12731764	1	66697753	G	A	0.266871	0.123513	0.00929875	2.91E-40	176.43

eTable 4. MR association between drug target gene expression in blood and inflammatory factor levels(Psmr_threshold<0.05) (Continued)

Probe ID	Inflammatory factors association			MR association (changes per 1SD increase in expression)			HEIDI Test		Phenotype	eQTL Samplesize	Variances (R2) in the exposure explained by genetic instruments	Power(%)
	beta	se	p-value	beta	se	p-value	p-value	Number of SNPs				
ENSG00000137752	-0.007482	0.00372	0.25924	-0.0787263	0.0397055	0.04739439	0.04634046	8	CRP	31684	0.066296761	99.2
ENSG00000110092	-0.00804	0.003764	0.22764	0.0633915	0.029946	0.03427199	0.8453412	5	CRP	31684	0.088797328	100
ENSG00000204386	0.019026	0.003989	0.0032606	0.277293	0.0762638	0.000276933	0.634121	6	CRP	31684	0.03155609	78
ENSG00000183134	0.007999	0.003983	0.2592	0.0703067	0.0354411	0.04728252	0.6358919	10	CRP	31684	0.071442466	100
ENSG00000075239	-0.008441	0.003632	0.18348	-0.0223552	0.00964386	0.02044544	0.4137433	5	CRP	31684	0.179989562	100
ENSG00000059377	-0.010668	0.004049	0.12618	-0.0692249	0.0265141	0.009031215	NA	NA	CRP	31684	0.10892818	100
ENSG00000151623	-0.011765	0.003813	0.67855	0.160423	0.0549527	0.003508187	0.1178453	4	CRP	31684	0.050624628	83.6
ENSG00000182718	0.009479	0.003588	0.12457	0.102711	0.0399134	0.01007193	NA	NA	CRP	31684	0.063833147	98.6
ENSG00000185245	0.0419	0.0177	0.01798	0.431094	0.187503	0.02149759	0.214005	20	IL6	31684	0.05420158	98.1
ENSG00000012223	0.0413	0.0174	0.01634	0.797848	0.371369	0.0316822	0.1650111	20	IL6	31684	0.030760377	36.5
ENSG00000144852	0.0346	0.0163	0.03402	-0.6694	0.33529	0.04588169	0.1057458	20	IL6	31684	0.03301087	38.7
ENSG00000164111	0.043	0.0216	0.04787	0.0543372	0.0273024	0.04656889	0.0147197	20	IL6	31684	NA	NA
ENSG00000128602	0.0445	0.0204	0.02931	-0.487316	0.230111	0.0341972	0.0205939	20	IL6	31684	0.049588313	95.2
ENSG00000044574	0.456062	0.028771	1.37048E-56	1.56653	0.58764	0.00768	0.004127	19	TNF	31684	0.032467172	100
ENSG00000132170	0.252822	0.007947	4.0925E-222	-0.458262	0.163949	0.005188	0.218256	20	TNF	31684	0.034876483	97.1
ENSG00000213903	-0.14731	0.008347	1.03209E-69	0.658142	0.320171	0.039822	0.454903	20	TNF	31684	0.035149954	89.9
ENSG00000136997	0.137118	0.0533095	0.01010802	-1.71683	0.695443	0.01356093	0.3026708	20	IL1_beta	31684	0.049377739	88.5

ENSG00000151012	0.19318	0.0720535	0.007338862	1.97221	0.771047	0.01053278	0.5729089	12	IL1_beta	31684	0.047924498	96.8
ENSG00000184588	0.14481	0.0720675	0.04449831	1.17242	0.590117	0.04694879	0.5543008	20	IL1_beta	31684	0.07452206	100

The SNP instrument for each drug target gene is the SNP with the most significant association with gene expression in blood (top eQTL SNP). The table shows 1. Association of the SNP instrument with gene expression in the blood (eQTL association) in the eQTLGen consortium, where beta is the standard deviation change in gene expression per coded allele; 2. Association of the SNP instrument with inflammatory factors level in the Ligthart et al, Folkersen et al, and Gilly et al genome-wide association study, where beta is the change in inflammatory factors level in blood per coded allele; 3. MR association between gene expression and inflammatory factors level, where beta is the change in inflammatory factors level per 1 standard deviation increase in gene expression. 4. A significant HEIDI p-value (<0.05) indicates that any association between gene expression and the outcome may be due to linkage where there are 2 distinct causal variants in linkage disequilibrium (LD). Derivation of the HEIDI p-value can only be done if a sufficient number of SNPs (5 or more SNPs required) are available in the region that has 0.05 < LD r-squared < 0.9 with the top eQTL SNP. Note that the MR effects reported in this table are not harmonised based on the direction of effect of gene expression on inflammatory factors level in blood but simply report the effect on disease risk per 1SD increase in gene expression. Abbreviations: se, standard error; SNP, single nucleotide polymorphism; eQTL, expression quantitative trait loci; MR, mendelian randomisation; HEIDI, Heterogeneity in dependent instruments.

eTable 5. MR association between drug target gene expression in blood and MDD (Psmr_threshold < 0.0006578[0.05/19/4])

Probe ID	Gene	Chromosome	Base pair	top eQTL SNP	SNP Chromosome	SNP base pair	coded allele	other allele	coded allele frequency	eQTL association			
										beta	se	p-value	F-statistic
ENSG00000137752	CASP1	11	104934164	rs2409065	11	105063849	G	A	0.0715746	0.476604	0.0147974	1.33E-227	1037.40
ENSG00000110092	CCND1	11	69462548	rs1960217	11	69383728	C	T	0.362986	-0.184464	0.00909432	1.80E-91	411.42
ENSG00000204386	NEU1	6	31828059	rs367364	6	32019946	T	C	0.110429	-0.146845	0.0177525	1.32E-16	68.42
ENSG00000183134	PTGDR2	11	60620928	rs530963	11	60617834	C	A	0.448875	0.113773	0.00893556	3.90E-37	162.12
ENSG00000075239	ACAT1	11	108005373	rs4550189	11	107986344	G	A	0.417178	0.377586	0.0116896	6.77E-229	1043.36
ENSG00000059377	TBXAS1	7	139598487	rs2240395	7	139718147	G	C	0.354806	-0.284525	0.00813432	4.81E-268	1223.48
ENSG00000151623	NR3C2	4	149182881	rs6817545	4	148977389	T	C	0.429448	-0.0765647	0.00815629	6.16E-21	88.12
ENSG00000182718	ANXA2	15	60667207	rs62004990	15	60686631	T	C	0.273006	0.28109	0.00918197	8.17E-206	937.17
ENSG00000185245	GP1BA	17	4836958	rs67059207	17	4869093	C	T	0.210634	0.0971946	0.0100672	4.70E-22	93.21
ENSG00000012223	LTF	3	46501930	rs181225830	3	46369762	A	G	0.0235174	-0.344774	0.0487521	1.53E-12	50.01
ENSG00000144852	NR1I2	3	119518331	rs3732357	3	119530858	G	A	0.267894	-0.0516881	0.00879438	4.17E-09	34.54
ENSG00000164111	ANXA5	4	122603689	rs28454132	4	122613459	A	C	0.176892	0.81509	0.00908192	0	8054.82

ENSG00000128602	SMO	7	128841049	rs2718102	7	128810971	T	C	0.194274	-0.0907668	0.010248	8.22E-19	78.45
ENSG00000044574	HSPA5	9	128000370	rs2416962	9	128118864	G	A	0.02045	0.456062	0.028771	1.37E-56	251.27
ENSG00000132170	PPARG	3	12402361	rs1699346	3	12504295	C	A	0.429448	0.252822	0.007947	4.09E-222	1012.10
ENSG00000213903	LTB4R	14	24783949	rs3181256	14	24800879	G	A	0.317996	-0.14731	0.008347	1.03E-69	311.46
ENSG00000136997	MYC	8	128750677	rs10956401	8	129002419	A	G	0.382413	-0.0808447	0.00909205	6.01E-19	79.06
ENSG00000151012	SLC7A11	4	139124377	rs28364685	4	139164388	G	A	0.0460123	0.216242	0.0196773	4.30E-28	120.77
ENSG00000184588	PDE4B	1	66549228	rs12731764	1	66697753	G	A	0.266871	0.123513	0.00929875	2.91E-40	176.43

eTable 5. MR association between drug target gene expression in blood and MDD (Psmr_threshold < 0.0006578[0.05/19/4] (Continued)

Probe ID	GWAS association			MR association (log odds per 1SD increase in expression)			HEIDI Test		Phenotype	eQTL Samplesize	Variances (R2) in the exposure explained by genetic instruments	Power(%)
	beta	se	p-value	beta	se	p-value	p-value	Number of SNPs				
ENSG00000137752	-0.0008	0.0086	0.9285	-0.00167854	0.0180444	0.9258854	0.1979594	17	CRP	31684	0.179483988	100
ENSG00000110092	-0.0056	0.0045	0.2193	0.0303583	0.0244409	0.2141964	0.2012364	20	CRP	31684	0.113587561	100
ENSG00000204386	-0.0316	0.0058	6.27E-08	0.215192	0.0472951	5.36E-06	0.2945077	20	CRP	31684	0.046447293	100
ENSG00000183134	0.0052	0.0044	0.232	0.0457051	0.0388398	0.2392913	0.6706365	20	CRP	31684	0.071442466	100
ENSG00000075239	0.0009	0.0044	0.8369	0.00238356	0.0116532	0.8379305	0.2827902	20	CRP	31684	0.179989562	100
ENSG00000059377	-0.0118	0.0045	0.009401	0.0414727	0.0158602	0.008925761	0.02619435	20	CRP	31684	0.194632915	100
ENSG00000151623	0.0046	0.0044	0.301	-0.0600799	0.057823	0.2987905	0.02122176	20	CRP	31684	0.052702665	87.9
ENSG00000182718	0.0078	0.005	0.1188	0.0277492	0.017811	0.1192384	0.5192327	20	CRP	31684	0.17072686	85.3
ENSG00000185245	-0.0016	0.0055	0.7673	-0.0164618	0.0566132	0.7712218	0.7576281	20	IL6	31684	0.05420158	98.1
ENSG00000012223	-0.0029	0.0189	0.8797	0.0084113	0.0548313	0.8780804	0.7341689	7	IL6	31684	0.039714785	100

ENSG00000144852	-0.0069	0.0048	0.1498	0.133493	0.095602	0.1626115	0.01498628	20	IL6	31684	0.03301087	38.1
ENSG00000164111	0.0002	0.0054	0.9707	0.000245372	0.00662504	0.9704555	0.367671	20	IL6	31684	NA	100
ENSG00000128602	0.0082	0.0054	0.129	-0.0903414	0.0603612	0.1344764	0.3465132	20	IL6	31684	0.049729576	98
ENSG00000044574	-0.0429	0.0147	0.003564	-0.094066	0.032774	0.004103	0.605302	18	TNF	31684	0.088881267	100
ENSG00000132170	-0.0008	0.0044	0.857	-0.003164	0.017404	0.855728	0.231189	20	TNF	31684	0.177321565	100
ENSG00000213903	0.0038	0.0046	0.4103	-0.025796	0.031261	0.409267	0.569727	20	TNF	31684	0.098914117	100
ENSG00000136997	-0.0033	0.0045	0.4694	0.040819	0.0558513	0.4648696	0.5901997	20	IL1_beta	31684	0.049925029	89.5
ENSG00000151012	-0.0039	0.0112	0.7316	-0.0180354	0.0518199	0.7278104	0.2095251	20	IL1_beta	31684	0.061681579	100
ENSG00000184588	-0.0141	0.005	0.004642	-0.114158	0.0413837	0.00580642	0.002103729	20	IL1_beta	31684	0.07452206	100

The SNP instrument for each drug target gene is the SNP with the most significant association with gene expression in the blood (top eQTL SNP). The table shows 1. Association of the SNP instrument with gene expression in the blood (eQTL association), where beta is the standard deviation change in gene expression per coded allele; 2. Association of the SNP instrument with major depressive disorder risk in the Wary et al genome-wide association study, where beta is the log odds ratio per coded allele 3. MR association between gene expression and major depressive disorder, where beta is the log odds per 1 standard deviation increase in gene expression; 4. A significant HEIDI p-value (<0.05) indicates that any association between gene expression and outcome may be due to linkage where there are 2 distinct causal variants in linkage disequilibrium (LD). Derivation of the HEIDI p-value can only be done if a sufficient number of SNPs (5 or more SNPs required) are available in the re gion that has 0.05 < LD r-squared < 0.9 with the top eQTL SNP. Note that the MR effects reported in this table are not harmonised based on the direction of effect of gene expression on inflammatory factors level in

blood but simply report the effect on disease risk per 1SD increase in gene expression. Abbreviations: se, standard error; SNP, single nucleotide polymorphism; eQTL, expression quantitative trait loci; MR, mend elian randomisation; HEIDI, Heterogeneity in dependent instruments.

eTable 6. MR association between expression in blood of genes adjactent to NEU1 and MDD risk(Psmr_threshold < 0.00116[0.05/43])

Probe ID	Gene	Gene Chromosome	Gene start position (hg19)	eQTL SNP	SNP Chromosome	SNP hg19 position	base pair distance of gene from SNP	coded allele	other allele	coded allele frequency	eQTL association			
											beta	se	p-value	F-statistics
ENSG00000204386	NEU1	6	31828059	rs367364	6	32019946	32822975	T	C	0.110429	-0.146845	0.0177525	1.32E-16	68.42
ENSG00000204444	APOM	6	31623090	rs693906	6	31835164	31835163.99	C	G	0.112474	-0.14626	0.0175945	9.35E-17	69.10
ENSG00000168477	TNXB	6	32046021	rs2269426	6	32076499	32076499	A	G	0.41002	0.434818	0.0181727	1.60E-126	572.50
ENSG00000204389	HSPA1A	6	31784504	rs2523568	6	31329932	31329931.99	A	G	0.175869	0.0890286	0.0188261	2.26E-06	22.36
ENSG00000204469	PRRC2A	6	31597022	rs707939	6	31726688	31726688	A	C	0.368098	0.166081	0.0159942	2.94E-25	107.82
ENSG00000213676	ATF6B	6	32080991	rs501942	6	31840477	31840476.99	T	C	0.0715746	0.347927	0.0248494	1.53E-44	196.04
ENSG00000213760	ATP6V1G2	6	31514221	rs2523502	6	31513864	31513863.99	A	T	0.174847	-0.268628	0.015728	2.11E-65	291.71
ENSG00000204314	PRRT1	6	32119143	rs570263	6	31847196	31847196	T	C	0.276074	0.0930306	0.0131992	1.81E-12	49.68
ENSG00000221988	PPT2	6	32126336	rs553108	6	31840455	31840455	A	G	0.374233	0.169623	0.0157271	4.03E-27	116.32
ENSG00000206337	HCP5	6	31406881	rs1800628	6	31546850	32577692.99	A	G	0.0777096	-0.246199	0.022139	9.96E-29	123.67
ENSG00000198563	DDX39B	6	31504110	rs3093949	6	31525184	32589771	T	C	0.309816	-0.335868	0.0168453	1.89E-88	397.54
ENSG00000204435	CSNK2B	6	31635566	rs1144708	6	31710020	31710020	T	C	0.366053	0.177755	0.0124444	2.75E-46	204.03
ENSG00000204520	MICA	6	31377224	rs149692329	6	31385843	31385842.99	T	C	0.224949	-0.749615	0.0170557	0	1931.69
ENSG00000204463	BAG6	6	31613643	rs144802800	6	31499402	31499401.99	A	G	0.0664622	0.269138	0.0289676	1.53E-20	86.32
ENSG00000204536	CCHCR1	6	31118115	rs3130573	6	31106268	31106268	G	A	0.346626	0.291928	0.0123143	3.10E-124	561.99
ENSG00000229391	HLA-DRB6	6	32524144	rs112112734	6	32453853	32453853	C	T	0.43865	-0.72183	0.00873192	0	6833.61
ENSG00000204315	FKBPL	6	32097276	rs17840121	6	32577693	32577692.99	C	G	0.150307	0.173624	0.0192266	1.71E-19	81.55
ENSG00000228789	HCG22	6	31024447	rs1265054	6	31079643	32453853	C	T	0.510225	-0.46156	0.020001	7.92E-118	532.54

ENSG00000241404	EGFL8	6	32134209	rs41268896	6	32070069	32577692.99	A	G	0.343558	-0.122447	0.0128212	1.29E-21	91.21
ENSG00000237541	HLA-DQA1	6	32712055	rs9271520	6	32589771	32589771	G	A	0.291411	1.1376	0.015154	0	5635.40
ENSG00000204252	HLA-DOA	6	32974672	rs381218	6	32977420	31535074	T	G	0.252556	-0.286034	0.0139662	3.22E-93	419.45
ENSG00000234745	HLA-B	6	31323307	rs2596496	6	31322782	31322782	C	G	0.309816	0.640329	0.0160476	0	1592.16
ENSG00000204356	NELFE	6	31923375	rs541862	6	31916951	31916950.99	C	T	0.0879346	-0.462702	0.0298006	2.29E-54	241.08
ENSG00000204438	GPANK1	6	31631533	rs139938004	6	31770134	31770133.99	A	G	0.0286299	0.490879	0.0353298	6.87E-44	193.05
ENSG00000204351	SKIV2L	6	31932194	rs389512	6	31947594	31947593.99	C	G	0.140082	0.737882	0.0177404	0	1730.00
ENSG00000204472	AIF1	6	31583879	rs142831262	6	31576714	31576713.99	A	G	0.161554	-0.317785	0.0170794	2.85E-77	346.20
ENSG00000179344	HLA-DQB1	6	32631702	rs9274660	6	32636434	32636434	A	G	0.407975	0.644563	0.00904771	0	5075.20
ENSG00000232810	TNF	6	31544728	rs1121800	6	31535074	31535074	A	T	0.40593	0.297085	0.0121342	2.23E-132	599.43
ENSG00000204261	PSMB8-AS1	6	32813067	rs3763347	6	32822975	32822975	A	T	0.397751	-0.407695	0.0198451	8.74E-94	422.05
ENSG00000166278	C2	6	31889505	rs488755	6	31904306	31535074	A	G	0.0664622	-0.255966	0.0269983	2.52E-21	89.89
ENSG00000240065	PSMB9	6	32819637	rs2071477	6	32825348	32825348	C	T	0.397751	0.490188	0.0115773	0	1792.71
ENSG00000198502	HLA-DRB5	6	32491592	rs9271055	6	32575369	32575368.99	G	T	0.139059	1.18659	0.00941556	0	15882.14
ENSG00000204257	HLA-DMA	6	32926630	rs1050391	6	32917857	32917856.99	A	G	0.0674847	0.359763	0.0235827	1.52E-52	232.73
ENSG00000204525	HLA-C	6	31238216	rs9264547	6	31234742	31234741.99	C	G	0.194274	0.735868	0.00979082	0	5648.87
ENSG00000240053	LY6G5B	6	31639748	rs1144709	6	31709349	31709348.99	T	C	0.239264	-0.294717	0.0140732	2.23E-97	438.56
ENSG00000241106	HLA-DOB	6	32782682	rs3763354	6	32786917	32786916.99	G	A	0.202454	0.635461	0.0142786	0	1980.64
ENSG00000204531	POU5F1	6	31139380	rs35303934	6	31234222	31535074	A	G	0.127812	0.849835	0.0256166	2.45E-241	1100.59
ENSG00000204387	SNHG32	6	31804963	rs140091914	6	31754008	31754007.99	A	G	0.156442	-0.446256	0.0169078	1.63E-153	696.62
ENSG00000204308	RNF5	6	32149030	rs62402717	6	32080838	32080837.99	A	G	0.0368098	0.655614	0.0629587	2.15E-25	108.44
ENSG00000204392	LSM2	6	31769967	rs34814308	6	31777641	31777640.99	T	C	0.0296524	-0.770798	0.0444551	2.40E-67	300.63
ENSG00000204348	DXO	6	31938828	rs17201560	6	32047268	32047267.99	C	T	0.0368098	-0.435137	0.0403622	4.24E-27	116.23

ENSG00000204498	NFKBIL1	6	31520626	rs116282626	6	31515619	31515618.99	A	G	0.0572597	-0.252979	0.0283641	4.71E-19	79.55
ENSG00000204267	TAP2	6	32798083	rs4148876	6	32796793	32796792.99	A	G	0.0756646	-1.19718	0.0338545	6.40E-274	1250.51

eTable 6. MR association between expression in blood of genes adjactent to NEU1 and MDD risk(Psmr_threshold < 0.00116[0.05/43]) (Continued)

Probe ID	MDD association			MR association (log odds per 1SD increase in expression)			HEIDI Test		Posterior probability for shared variant for gene expression and MDD risk
	beta	se	p-value	beta	se	p-value	p-value	Number of SNPs	
	-0.0316	0.0058	6.27E-08	0.215192	0.0472951	5.36E-06	0.2945077	20	1.0000
ENSG00000204386	-0.0322	0.0061	1.39E-07	0.220156	0.049405	8.34E-06	0.126918	20	<0.0000001
ENSG00000204444	0.0191	0.0045	1.90E-05	0.0439264	0.0105107	2.93E-05	0.1728316	20	<0.0000001
ENSG00000168477	-0.0314	0.0052	1.16E-09	-0.352695	0.0947307	0.000196762	0.4018253	8	<0.0000001
ENSG00000204389	0.0147	0.0046	0.001254	0.088511	0.0289793	0.002255979	0.05428422	20	<0.0000001
ENSG00000204469	-0.0405	0.0066	1.03E-09	-0.116404	0.0207114	1.91E-08	0.00723865	20	<0.0000001
ENSG00000213676	-0.0148	0.0054	0.006273	0.0550948	0.0203593	0.006807431	0.02459665	20	<0.0000001
ENSG00000213760	-0.0181	0.0045	6.45E-05	-0.19456	0.0556934	0.000476908	0.03688417	20	<0.0000001
ENSG00000204314	-0.0172	0.0044	9.08E-05	-0.101401	0.0275911	0.000237715	0.00322059	20	<0.0000001
ENSG00000221988	-0.043	0.0065	3.25E-11	0.174655	0.0307197	1.30E-08	0.009500643	20	<0.0000001
ENSG00000206337	-0.0169	0.0045	0.0001742	0.0503174	0.0136337	0.000223676	2.23E-08	20	<0.0000001
ENSG00000198563	-0.0222	0.0045	8.56E-07	-0.124891	0.026783	3.12E-06	0.003373116	20	<0.0000001
ENSG00000204435	0.0145	0.0057	0.01057	-0.0193433	0.00761663	0.01109764	0.000872277	20	<0.0000001
ENSG00000204520	0.0248	0.0094	0.008309	0.0921462	0.0363072	0.01115015	0.370416	20	<0.0000001
ENSG00000204463	-0.0108	0.0046	0.01817	-0.0369955	0.0158344	0.01947062	0.01531332	20	<0.0000001

ENSG00000204536	0.0104	0.0045	0.02103	-0.0144078	0.00623659	0.02087662	0.4199371	20	<0.0000001
ENSG00000229391	0.0138	0.0061	0.02325	0.0794822	0.0362191	0.02820093	0.1601999	20	<0.0000001
ENSG00000204315	-0.0088	0.0043	0.04096	0.0190658	0.0093528	0.04149883	5.27E-05	20	<0.0000001
ENSG00000228789	0.0093	0.0047	0.04695	-0.0759511	0.0391991	0.05267527	0.002853461	20	<0.0000001
ENSG00000241404	0.0076	0.0046	0.09781	0.00668075	0.00404459	0.09858065	0.03046867	20	<0.0000001
ENSG00000237541	0.0074	0.0049	0.1324	-0.025871	0.0171773	0.1320377	0.00919034	20	<0.0000001
ENSG00000204252	0.0069	0.0046	0.1344	0.0107757	0.00718888	0.1338889	8.76E-09	20	<0.0000001
ENSG00000234745	0.011	0.0074	0.1341	-0.0237734	0.0160661	0.1389478	0.04110084	20	<0.0000001
ENSG00000204356	0.0155	0.0106	0.1445	0.031576	0.0217132	0.1458818	0.3829212	20	<0.0000001
ENSG00000204438	0.0076	0.0064	0.2313	0.0102997	0.00867701	0.2352212	0.02127627	20	<0.0000001
ENSG00000204351	-0.0072	0.0064	0.261	0.0226568	0.0201762	0.2614591	0.004669459	20	<0.0000001
ENSG00000204472	0.0045	0.0044	0.3081	0.00698148	0.00682704	0.3064867	0.997646	20	<0.0000001
ENSG00000179344	0.0045	0.0044	0.3112	0.0151472	0.0148235	0.3068585	7.76E-06	20	<0.0000001
ENSG00000232810	-0.0039	0.0044	0.3693	0.00956598	0.0108024	0.3758656	0.1309266	20	<0.0000001
ENSG00000204261	0.0071	0.0083	0.3885	-0.0277381	0.0325579	0.3942358	0.2487511	20	<0.0000001
ENSG00000166278	-0.0037	0.0044	0.3945	-0.00754812	0.00897791	0.4004918	0.1998146	20	<0.0000001
ENSG00000240065	-0.0045	0.0059	0.4499	-0.00379237	0.00497231	0.4456436	0.9403372	20	<0.0000001
ENSG00000198502	-0.0049	0.0072	0.4953	-0.0136201	0.0200331	0.4965808	0.01246104	20	<0.0000001
ENSG00000204257	-0.0035	0.0053	0.5105	-0.00475629	0.00720265	0.5090281	5.69E-07	20	<0.0000001
ENSG00000204525	-0.0032	0.0053	0.5505	0.0108579	0.0179908	0.5461609	5.39E-06	20	<0.0000001
ENSG00000240053	-0.0031	0.0052	0.5537	-0.00487835	0.00818377	0.5511082	0.002244677	20	<0.0000001
ENSG00000241106	0.0034	0.0063	0.5826	0.00400077	0.00741418	0.5894652	0.01806138	20	<0.0000001
ENSG00000204531	-0.0017	0.0067	0.7946	0.00380948	0.0150145	0.7997122	9.97E-06	20	<0.0000001

ENSG00000204387	0.0025	0.012	0.8313	0.00381322	0.0183071	0.8350012	0.09291928	20	<0.0000001
ENSG00000204308	0.0025	0.0149	0.8688	-0.00324339	0.0193315	0.8667584	0.3723408	20	<0.0000001
ENSG00000204392	-0.0018	0.0109	0.872	0.00413663	0.0250525	0.8688509	0.2590568	20	<0.0000001
ENSG00000204348	0.0015	0.0133	0.9091	-0.00592934	0.0525777	0.9102106	0.07104789	20	<0.0000001
ENSG00000204498	-1.00E-04	0.0084	0.9866	8.35E-05	0.00701646	0.9905016	0.03850887	20	<0.0000001
ENSG00000204267	-1.00E-04	0.0084	0.9866	8.35E-05	0.00701646	0.9905016	0.03850887	20	<0.0000001

The SNP instrument for each gene that is nominally associated with the top eQTL for NEU1 in blood is the SNP with the most significant association with gene expression in blood (top eQTL SNP). The table shows 1. Association of the SNP instrument with gene expression in blood (eQTL association), where beta is the standard deviation change in gene expression per coded allele; 2. Association of the SNP instrument with major depressive disorder in the Wary et al genome-wide association study, where beta is the log odds per coded allele 3. MR association between gene expression and major depressive disorder, where beta is the log odds per 1 standard deviation increase in gene expression; 4. A significant HEIDI p-value (<0.05) indicates that any association between gene expression and outcome may be due to linkage where there are 2 distinct causal variants in linkage disequilibrium (LD); 5. The posterior probability from coloc analysis for a shared causal variant that affects both expression and outcome. Derivation of the HEIDI p-value can only be done if a sufficient number of SNPs (5 or more SNPs required) are available in the region that have 0.05 < LD r-squared < 0.9 with the top eQTL SNP. Note that the MR effects reported in this table are not harmonised based on the direction of effect of gene expression on inflammatory factor levels but simply report the effect on disease risk per 1SD increase in gene expression. Abbreviations: se, standard error; SNP, single nucleotide polymorphism; eQTL, expression quantitative trait loci; MDD, major depressive disorder; MR, mendelian randomisation; HEIDI, Heterogeneity in dependent instruments.

eTable 7. Validated MR association between drug target gene expression in blood and inflammatory factor levels (Psmr_threshold < 0.05)

Probe ID	Gene	Chromosome	Base pair	top eQTL SNP	SNP Chromosome	SNP base pair	coded allele	other allele	coded allele frequency	GWAS association		
										beta	se	p-value
ILMN_1763144	NEU1	6	31827313	rs2734335	6	31893944	G	A	0.48364	0.015865	0.003726	2.09E-05
ILMN_1662795	CA2	8	86393199	rs2930553	8	86849171	T	G	0.489775	0.007399	0.003544	0.0368553
ILMN_2199439	CA2	8	86393306	rs2930553	8	86849171	T	G	0.489775	0.007399	0.003544	0.0368553
ILMN_2282641	TBXAS1	7	139717597	rs3801148	7	139694500	C	T	0.493865	0.010668	0.004049	0.008438009
ILMN_2409167	ANXA2	15	60639822	rs3068	15	60715643	C	T	0.458078	-0.010975	0.003769	0.003599979
ILMN_1711899	ANXA2	15	60689484	rs7165874	15	60784609	A	T	0.43047	0.010141	0.003581	0.004637031
ILMN_2390457	ESR2	14	64701713	rs6573624	14	66226885	A	G	0.46319	0.010596	0.003626	0.003488028
ILMN_1787576	CLCNKA	1	16360510	rs77171346	1	14678082	C	T	0.0214724	-0.152	0.0617	0.01268
ILMN_2288976	PDE4B	1	66258222	rs12142975	1	67345082	C	T	0.192229	0.0567	0.0206	0.005721
ILMN_2323338	NR1I2	3	119537036	rs7612975	3	120413158	G	A	0.0460123	0.0878	0.0349	0.01234
ILMN_1741003	ANXA5	4	122589570	rs28651243	4	122618166	G	A	0.176892	0.0509	0.0232	0.02928
ILMN_1773865	HSPA5	9	127997213	rs17336754	9	127865325	G	C	0.0173824	0.576784	0.210628	0.006368082
ILMN_1800225	PPARG	3	12475678	rs1699346	3	12504295	C	A	0.429448	-0.115859	0.0412897	0.005304964
ILMN_1653793	PDPK1	16	2615677	rs149517429	16	2698418	C	T	0.153374	-0.107386	0.043042	0.01292429
ILMN_2323338	NR1I2	3	119537036	rs7612975	3	120413158	G	A	0.0460123	-0.375949	0.122759	0.002325367
ILMN_2110908	MYC	8	128753287	rs4733812	8	128999640	A	G	0.383436	0.137118	0.0533095	0.01010802
ILMN_1772399	ALOX5	10	45871187	rs2505747	10	44732332	A	G	0.407975	-0.175815	0.0584161	0.002615049
ILMN_1710590	PLA2G2E	1	20246872	rs12116775	1	21640501	A	G	0.0357873	-0.480857	0.167486	0.004091392

eTable 7. Validated MR association between drug target gene expression in blood and inflammatory factor levels (Psmr_threshold < 0.05) (Continued)

Probe ID	eQTL association				MR association (log odds per 1SD increase in expression)			HEIDI Test		Phenotype	eQTL Samplesize	Variances (R2) in the exposure explained by genetic instruments	Power(%)
	beta	se	p-value	F-statistic	beta	se	p-value	p-value	Number of SNPs				
ILMN_1763144	-0.0942171	0.0270237	4.89455E-16	12.15541646	0.168388	0.0624229	0.006985586	NA	NA	CRP	2765	0.284567351	75.3
ILMN_1662795	0.455369	0.0275899	3.38E-61	272.4122738	0.0162484	0.00784472	0.03833551	0.2437004	9	CRP	2765	0.306436603	100
ILMN_2199439	0.445883	0.0276137	1.19E-58	260.7309724	0.016594	0.00801443	0.03840377	0.2442414	10	CRP	2765	0.300105028	100
ILMN_2282641	-0.173049	0.0281107	7.46E-10	37.8961305	-0.0616473	0.0254509	0.01542685	NA	NA	CRP	2765	0.116723937	87.5
ILMN_2409167	0.136746	0.0277099	8.02E-07	24.35338564	-0.0802583	0.0320026	0.01214608	NA	NA	CRP	2765	0.093684693	59.5
ILMN_1711899	0.294454	0.0276564	1.80E-26	113.3557673	0.03444	0.0125843	0.006205197	NA	NA	CRP	2765	0.200509934	100
ILMN_2390457	-0.073009	0.026405	5.69282E-09	7.645052221	-0.145133	0.0722621	0.04459839	NA	NA	CRP	2765	0.05257007	14
ILMN_1787576	-0.266256	0.0803142	9.15842E-08	10.99041599	0.570879	0.288709	0.04800212	0.8386658	3	IL6	2765	0.063012099	94
ILMN_2288976	0.140696	0.039749	4.00717E-08	12.52884645	0.402997	0.185472	0.02979431	NA	NA	IL6	2765	0.067268535	48.4
ILMN_2323338	-0.234744	0.069677	7.54329E-10	11.35037249	-0.374024	0.18555	0.04382443	NA	NA	IL6	2765	0.064033574	87.8
ILMN_1741003	1.04096	0.0343115	3.56E-202	920.4255677	0.0488972	0.0223453	0.02865132	0.08022669	20	IL6	2765	0.53230534	100
ILMN_1773865	0.749611	0.118961	2.95E-10	39.70659894	0.769444	0.306369	0.0120222	0.00452278	9	TNF	2765	0.119460801	100
ILMN_1800225	0.227452	0.027238	6.80E-17	69.73151307	-0.509376	0.191506	0.007817691	0.1327727	20	TNF	2765	0.15787856	99.7
ILMN_1653793	0.196281	0.0420234	3.00E-06	21.81595101	-0.547102	0.248611	0.02776173	0.3834352	8	TNF	2765	0.088692059	86.7
ILMN_2323338	-0.234744	0.069677	7.54329E-09	11.35037249	1.60153	0.706719	0.02344233	NA	NA	TNF	2765	0.064033574	87.8
ILMN_2110908	-0.156931	0.0294331	9.73E-08	28.42794539	-0.873747	0.377162	0.02052363	0.6967618	9	IL1_beta	2765	0.101180645	74.7

ILMN_1772399	0.100911	0.027253	2.13277E-10	13.71034513	-1.74228	0.745999	0.01951757	0.4244254	5	IL1_beta	2765	0.070361342	29
ILMN_1710590	-0.247103	0.065534	1.62851E-09	14.21748185	1.94598	0.851916	0.02235761			IL1_beta	2765	0.071647549	93.9

The SNP instrument for each drug target gene is the SNP with the most significant association with gene expression in the blood (top eQTL SNP). The table shows 1. Association of the SNP instrument with gene expression in the blood (eQTL association) in the CAGE eQTL summary data, where beta is the standard deviation change in gene expression per coded allele; 2. Association of the SNP instrument with inflammatory factors level in the Ligthart et al, Folkersen et al, and Gilly et al genome-wide association study, where beta is the change in inflammatory factors level in blood per coded allele; 3. MR association between gene expression and inflammatory factors level, where beta is the change in inflammatory factors level per 1 standard deviation increase in gene expression. 4. A significant HEIDI p-value (<0.05) indicates that any association between gene expression and the outcome may be due to linkage where there are 2 distinct causal variants in linkage disequilibrium (LD). Derivation of the HEIDI p-value can only be done if a sufficient number of SNPs (5 or more SNPs required) are available in the region that has 0.05 < LD r-squared < 0.9 with the top eQTL SNP. Note that the MR effects reported in this table are not harmonised based on the direction of effect of gene expression on inflammatory factors level in blood but simply report the effect on disease risk per 1SD increase in gene expression. Abbreviations: se, standard error; SNP, single nucleotide polymorphism; eQTL, expression quantitative trait loci; MR, mendelian randomisation; HEIDI, Heterogeneity in dependent instruments.

eTable 8. Validation of MR association between drug target gene expression in blood and MDD by using UK Biobank database(Psmr_threshold < 0.000694[0.05/18/4])

Probe ID	Gene	Chromosome	Base pair	top eQTL SNP	SNP Chromosome	SNP base pair	coded allele	other allele	coded allele frequency	GWAS association		
										beta	se	p-value
ILMN_1763144	NEU1	6	31827313	rs3130063	6	31558702	T	C	0.0797546	-0.0078968	0.0017308	5.06E-06
ILMN_1662795	CA2	8	86393199	rs2930553	8	86849171	T	G	0.489775	0.00033212	0.0011741	0.7772666
ILMN_2199439	CA2	8	86393306	rs2930553	8	86849171	T	G	0.489775	0.00033212	0.0011741	0.7772666
ILMN_2282641	TBXAS1	7	139717597	rs10242990	7	139730500	G	C	0.298569	-0.0046663	0.0013041	0.00034586
ILMN_2409167	ANXA2	15	60639822	rs2414672	15	60692646	A	G	0.306748	0.0019312	0.0013032	0.1383758
ILMN_1711899	ANXA2	15	60689484	rs2414672	15	60692646	A	G	0.306748	0.0019312	0.0013032	0.1383758
ILMN_2390457	ESR2	14	64701713	rs118014723	14	65728820	A	G	0.0163599	6.65E-05	0.0044545	0.9880923
ILMN_1787576	CLCNKA	1	16360510	rs114824062	1	18157319	T	C	0.0347648	-0.0037786	0.0030254	0.2116752
ILMN_2288976	PDE4B	1	66258222	rs12142975	1	67345082	C	T	0.192229	0.00081284	0.001729	0.6382635
ILMN_2323338	NR1I2	3	119537036	rs7612975	3	120413158	G	A	0.0460123	0.0030923	0.0039544	0.4342102
ILMN_1741003	ANXA5	4	122589570	rs28454132	4	122613459	A	C	0.176892	-6.42E-05	0.001462	0.9649749
ILMN_1773865	HSPA5	9	127997213	rs17336754	9	127865325	G	C	0.0173824	-0.010264	0.0038882	0.008296597
ILMN_1800225	PPARG	3	12475678	rs1699346	3	12504295	C	A	0.429448	-0.00071075	0.0012034	0.5547662
ILMN_1653793	PDPK1	16	2615677	rs11641077	16	2699304	T	C	0.152352	-0.0013376	0.0017356	0.4408796
ILMN_2323338	NR1I2	3	119537036	rs7612975	3	120413158	G	A	0.0460123	0.0030923	0.0039544	0.4342102
ILMN_1680618	MYC	8	128753037	rs10956403	8	129007207	G	A	0.381391	0.00022815	0.0012388	0.8538724
ILMN_1772399	SLC7A11	10	45871187	rs60685600	10	44681591	G	A	0.186094	0.0006189	0.0015587	0.6913214
ILMN_1710590	PDE4B	1	20246872	rs115068440	1	21644994	G	A	0.0357873	0.0013147	0.0031473	0.6761452

eTable 8. Validation of MR association between drug target gene expression in blood and MDD by using UK Biobank database(Psmr_threshold < 0.000694[0.05/18/4]) (Continued)

Probe ID	eQTL association				MR association (log odds per 1SD increase in expression)			HEIDI Test		Phenotype	eQTL Samplesize	Variances (R2) in the exposure explained by genetic instruments	Power (%)
	beta	se	p-value	F-statistic	beta	se	p-value	p-value	Number of SNPs				
ILMN_1763144	-0.234392	0.0438483	9.02E-09	28.5745927	0.0336906	0.0097082	0.000519834	0.6963878	20	CRP	2765	0.101440044	97.5
ILMN_1662795	0.455369	0.0275899	3.38E-61	272.4122738	0.000729343	0.00257873	0.7773068	0.2283378	20	CRP	2765	0.306436603	100
ILMN_2199439	0.445883	0.0276137	1.19E-58	260.7309724	0.000744859	0.00263361	0.7773082	0.2328474	20	CRP	2765	0.300105028	100
ILMN_2282641	-0.341626	0.0310224	3.34E-28	121.2693488	0.0136591	0.00401379	0.000666404	0.8295702	20	CRP	2765	0.2072382	100
ILMN_2409167	0.446301	0.0314687	1.18E-45	201.139847	0.00432712	0.0029359	0.1405174	0.9356012	20	CRP	2765	0.264992666	100
ILMN_1711899	0.608495	0.0313898	1.03E-83	375.7828283	0.00317373	0.00214793	0.1395203	0.7827166	20	CRP	2765	0.356624312	100
ILMN_2390457	0.387869	0.099679	9.98E-05	15.14128716	0.000171403	0.0114846	0.9880924	NA	NA	CRP	2765	0.073930511	100
ILMN_1787576	0.268262	0.0746174	3.24184E-08	12.9252244	-0.0140855	0.0119389	0.2380823	NA	NA	IL6	2765	0.068321889	95.8
ILMN_2288976	0.140696	0.039749	4.00717E-08	12.52884645	0.00577728	0.0123968	0.6411948	NA	NA	IL6	2765	0.107268535	67.8
ILMN_2323338	-0.234744	0.069677	7.54329E-09	11.35037249	-0.0131731	0.0172934	0.4462156	NA	NA	IL6	2765	0.104033574	97.8
ILMN_1741003	1.13917	0.0352372	2.77E-229	1045.139582	-5.64E-05	0.00128339	0.9649753	0.1259208	20	IL6	2765	0.561230305	100
ILMN_1773865	0.749611	0.118961	2.95E-10	39.70659894	-0.0136924	0.00562372	0.01490147	0.3784958	9	TNF	2765	0.119460801	100
ILMN_1800225	0.227452	0.027238	6.80E-17	69.73151307	-0.00312484	0.005304	0.555763	0.0982563	20	TNF	2765	0.15787856	99.7
ILMN_1653793	0.180442	0.0401856	7.12E-06	20.16203434	-0.00741291	0.00975925	0.4475072	0.3528467	6	TNF	2765	0.085272636	79.1
ILMN_2323338	-0.234744	0.069677	7.54329E-07	11.35037249	-0.0131731	0.0172934	0.4462156	NA	NA	TNF	2765	0.064033574	87.8
ILMN_1680618	-0.171322	0.028883	3.00E-09		-0.0013317	0.00723431	0.8539496	0.7166204	20	IL1_beta	2765	0.112496414	85.6
ILMN_1772399	-0.137788	0.036206	1.41422E-08		-0.00449168	0.0113737	0.6929034	0.1890412	7		2765	0.12312027	72

ILMN_1710590	-0.249231	0.0652	1.32073E-08		-0.00527503	0.0127032	0.6779581	0.4813249	3		2765	0.126321599	99.7

The SNP instrument for each drug target gene is the SNP with the most significant association with gene expression in the
blood (top eQTL SNP). The table shows 1. Association of the SNP instrument with gene expression in the blood (eQTL association) in the CAGE eQTL summary
data, where beta is the standard deviation change in gene expression per coded allele; 2. Association of the SNP instrument with major depression disorder
(UKB) risk in the Howard et al genome-wide association study, where beta is the log odds ratio per coded allele 3. MR association between gene expression and major depressive disorder
(UKB), where beta is the log odds per 1 standard deviation increase in gene expression; 4. A significant HEIDI p-value (<0.05) indicates that any association between gene expression and outcome may be due
to linkage where there are 2 distinct causal variants in linkage disequilibrium (LD). Derivation of the HEIDI p-value can only be done if a sufficient number of SNPs (5 or more SNPs required) are available in the
region that has 0.05 < LD r-squared < 0.9 with the top eQTL SNP. Note that the MR effects reported in this table are not harmonised based on the direction of effect of gene expression on inflammatory factors
level in
blood but simply report the effect on disease risk per 1SD increase in gene expression. Abbreviations: se, standard error; SNP, single nucleotide polymorphism; eQTL, expression quantitative trait loci; MR, me
ndelian randomisation; HEIDI, Heterogeneity in dependent instruments.

eTable 9. MR association between drug target gene DNA methylation in blood and MDD(Psmr_threshold < 0.0002272[0.05/55/4])

Probe ID	Gene	Chromosome	Probe bp	top SNP	SNP Chromosome	top SNP base pair	coded allele	other allele	coded allele frequency	mQTL association			
										beta	se	p-value	F-statistic
cg01406280	CCND1	11	69454447	rs2930975	11	69451837	C	G	0.4918	0.232162	0.0322946	6.53E-13	51.68
cg07295918	CCND1	11	69463131	rs117827876	11	69382922	T	A	0.0184	0.630394	0.136101	3.62E-06	21.45
cg09520904	CCND1	11	69462943	rs78334914	11	69253395	T	C	0.0757	0.469521	0.0620144	3.70E-14	57.32
cg12129983	CCND1	11	69462660	rs9344	11	69462910	A	G	0.4949	-0.346542	0.031591	5.35E-28	120.33
cg27021553	CCND1	11	69462783	rs11263441	11	69252000	A	G	0.0849	0.376047	0.061204	8.04E-10	37.75
cg00397479	NEU1	6	31831510	rs693906	6	31835164	C	G	0.1125	-0.267588	0.0418791	1.66E-10	40.83
cg19036153	NEU1	6	31830561	rs41267082	6	31830593	C	A	0.0307	-0.662126	0.0960838	5.53E-12	47.49
cg25560247	NEU1	6	31832173	rs613165	6	31834398	A	G	0.1973	-0.312305	0.0462606	1.47E-11	45.58
cg04873221	ACAT1	11	107992290	rs11212515	11	107997465	A	T	0.3374	-0.684508	0.0342115	4.68E-89	400.33
cg08152564	ACAT1	11	107992034	rs10890813	11	107987784	A	G	0.3364	0.219439	0.0350476	3.82E-10	39.20
cg14994056	ACAT1	11	107992015	rs10890813	11	107987784	A	G	0.3364	0.200488	0.0349074	9.28E-09	32.99
cg19829446	ACAT1	11	107992300	rs10890813	11	107987784	A	G	0.3364	-0.513685	0.0328428	3.84E-55	244.63
cg24079790	ACAT1	11	107993467	rs112087419	11	107998126	A	G	0.1585	-0.226495	0.0413095	4.18E-08	30.06
cg00445997	TBXAS1	7	139478495	rs141212865	7	139404666	C	A	0.2168	-0.274549	0.0397754	5.11E-12	47.64
cg03604364	TBXAS1	7	139705703	rs2267705	7	139696248	C	T	0.3374	0.305451	0.0346357	1.16E-18	77.77
cg06750564	TBXAS1	7	139637446	rs2284207	7	139640362	C	T	0.3661	-0.193134	0.0351754	4.01E-08	30.15
cg14116596	TBXAS1	7	139528673	rs6952322	7	139524753	A	G	0.4427	-0.411675	0.0314918	4.73E-39	170.89
cg18317554	TBXAS1	7	139498041	rs7459106	7	139439007	G	A	0.2168	0.380794	0.0413404	3.23E-20	84.85
cg24286200	TBXAS1	7	139481268	rs4132077	7	139420225	C	T	0.2157	-0.220895	0.0401575	3.78E-08	30.26

cg02072495	ANXA2	15	60689285	rs11071525	15	60689238	C	G	0.2761	0.354051	0.0376466	5.22E-21	88.45
cg03957109	ANXA2	15	60654693	rs8029375	15	60654671	G	A	0.2444	1.09272	0.030885	3.43E-274	1251.76
cg09533293	ANXA2	15	60689670	rs59233126	15	60691920	A	G	0.4744	0.226562	0.0315143	6.52E-13	51.68
cg09785377	ANXA2	15	60644157	rs35828699	15	60639256	T	C	0.3272	-1.33049	0.019634	0	4592.04
cg13313836	ANXA2	15	60687284	rs12440452	15	60687230	A	C	0.4162	-0.316861	0.0326589	2.95E-22	94.13
cg27554954	ANXA2	15	60691595	rs8033800	15	60689179	T	A	0.4192	-0.934225	0.0276842	1.23E-249	1138.78
cg04432599	GP1BA	17	4835608	rs3514	17	4801594	G	C	0.1636	0.36851	0.0453248	4.28E-16	66.10
cg04970434	GP1BA	17	4838096	rs2283568	17	4852305	A	G	0.1135	-0.316383	0.0504772	3.66E-10	39.29
cg07892449	GP1BA	17	4835627	rs67501853	17	4882332	A	G	0.0859	0.479395	0.0668072	7.19E-13	51.49
cg11340260	GP1BA	17	4835476	rs3514	17	4801594	G	C	0.1636	0.383518	0.0457935	5.53E-17	70.14
cg11902961	GP1BA	17	4835341	rs3514	17	4801594	G	C	0.1636	0.376466	0.0457695	1.95E-16	67.65
cg21934015	GP1BA	17	4834179	rs238245	17	4847443	T	C	0.2004	0.552786	0.0399523	1.54E-43	191.44
cg00716083	LTF	3	46506554	rs4682816	3	46487775	G	C	0.4315	0.379933	0.0324946	1.40E-31	136.71
cg04910591	LTF	3	46506104	rs4683234	3	46501769	G	C	0.3446	-0.259291	0.0350493	1.38E-13	54.73
cg10749572	LTF	3	46506864	rs4682816	3	46487775	G	C	0.4315	0.3079	0.0325175	2.83E-21	89.66
cg16975587	LTF	3	46506206	rs743659	3	46489015	G	A	0.3333	-0.277711	0.0349806	2.04E-15	63.03
cg17527798	LTF	3	46506404	rs4682816	3	46487775	G	C	0.4315	0.262134	0.0325964	8.85E-16	64.67
cg25930780	LTF	3	46506292	rs1042073	3	46484964	A	G	0.3344	-0.249027	0.0346423	6.55E-13	51.67
cg26307117	LTF	3	46506552	rs4682816	3	46487775	G	C	0.4315	0.428511	0.0320769	1.05E-40	178.46
cg27314002	LTF	3	46506519	rs4682816	3	46487775	G	C	0.4315	0.243645	0.0327922	1.09E-13	55.20
cg03354519	ANXA5	4	122618501	rs28651243	4	122618166	G	A	0.1769	-0.224195	0.0399395	1.98E-08	31.51
cg08715877	ANXA5	4	122618117	rs11098635	4	122586056	A	G	0.2444	-0.22146	0.0350917	2.77E-10	39.83
cg08831277	ANXA5	4	122617747	rs2306417	4	122617718	C	G	0.1115	-0.38035	0.0474192	1.05E-15	64.34

cg19501813	ANXA5	4	122618159	rs6857766	4	122611285	A	G	0.1779	0.727609	0.037861	2.62E-82	369.33
cg22097342	ANXA5	4	122618153	rs28454132	4	122613459	A	C	0.1769	0.380967	0.0397651	9.66E-22	91.78
cg20025238	SMO	7	128829789	rs13243618	7	128785008	C	T	0.2577	0.260784	0.0382564	9.31E-12	46.47
cg04632671	PPARG	3	12329826	rs11709077	3	12336507	A	G	0.1186	-0.348151	0.0492914	1.63E-12	49.89
cg23514324	PPARG	3	12329213	rs2920502	3	12329195	C	G	0.3180	0.547538	0.0339623	1.79E-58	259.92
cg04704294	PDE4B	1	66257822	rs11208743	1	66242820	T	C	0.327198	0.821612	0.0306521	2.87E-158	718.48
cg05694621	PDE4B	1	66767231	rs6683977	1	66769100	G	C	0.436605	0.992111	0.0259091	0	1466.28
cg08716655	PDE4B	1	66257732	rs12401401	1	66231308	A	G	0.327198	0.828167	0.0308486	9.35E-159	720.72
cg09320652	PDE4B	1	66708864	rs74531536	1	66711918	G	A	0.0245399	-2.83766	0.12481	1.98E-114	516.92
cg16294013	PDE4B	1	66258022	rs12034009	1	66255225	C	T	0.327198	0.556205	0.0335251	8.13E-62	275.25
cg22336004	PDE4B	1	66258035	rs12034009	1	66255225	C	T	0.327198	0.417714	0.033843	5.33E-35	152.34
cg22488256	PDE4B	1	66258046	rs11208743	1	66242820	T	C	0.327198	0.544605	0.0333677	6.96E-60	266.39
cg24058805	PDE4B	1	66777579	rs2180336	1	66777996	T	C	0.43865	0.351464	0.0321416	7.85E-28	119.57

eTable 9. MR association between drug target gene DNA methylation in blood and MDD(Psmr_threshold < 0.0002272[0.05/55/4]) (Continued)

Probe ID	GWAS association			MR association (log odds per 1SD increase in expression)			HEIDI Test		DNAm features		Phenotype
	beta	se	p-value	beta	se	p-value	p-value	Number of SNPs	feature	cgi	CRP
cg01406280	0.0089	0.0044	0.0412	0.0383353	0.0196882	0.05152034	0.6805864	20	TSS1500	island	CRP
cg07295918	-0.0057	0.0145	0.6956	-0.00904197	0.0230842	0.6952834	NA	NA	Body	shore	CRP
cg09520904	-0.0052	0.0082	0.5252	-0.0110751	0.0175258	0.5274303	0.6519909	13	Body	shore	CRP
cg12129983	0.0048	0.0044	0.2737	-0.0138511	0.0127595	0.2776762	0.01222593	20	Body	shore	CRP

cg27021553	-0.0056	0.008	0.4849	-0.0148918	0.0214116	0.4867415	0.3112998	14	Body	shore	CRP
cg00397479	-0.0322	0.0061	1.39E-07	-0.120334	0.0295694	4.71E-05	0.2524661	20	TSS1500	island	CRP
cg19036153	-0.022	0.0118	0.0623	0.0332263	0.0184621	0.07190763	0.2757801	20	TSS1500	island	CRP
cg25560247	-0.0011	0.006	0.8488	0.0035222	0.0192191	0.8545895	0.02562873	20	TSS1500	island	CRP
cg04873221	0.0033	0.0047	0.4826	-0.00482098	0.00687047	0.4828689	0.1770627	20	5'UTR	island	CRP
cg08152564	0.0034	0.0047	0.4655	0.0154941	0.0215607	0.4723728	0.3043782	20	TSS1500	island	CRP
cg14994056	0.0034	0.0047	0.4655	0.0169586	0.0236281	0.4729216	0.3695272	20	TSS1500	island	CRP
cg19829446	0.0034	0.0047	0.4655	-0.00661884	0.00915936	0.4699062	0.3225453	20	5'UTR	island	CRP
cg24079790	-0.0006	0.0056	0.913	0.00264907	0.0247294	0.9146919	0.4652333	20	Body	shore	CRP
cg00445997	-0.0097	0.0055	0.07448	0.0353307	0.0206764	0.08749887	0.9287255	20	5'UTR	island	CRP
cg03604364	-0.0013	0.0046	0.7832	-0.00425601	0.0150674	0.777588	0.086121	20	Body	opensea	CRP
cg06750564	0.0049	0.0046	0.2807	-0.025371	0.0242618	0.2956907	0.8306758	15	Body	opensea	CRP
cg14116596	-0.0002	0.0043	0.9564	0.000485821	0.0104452	0.9629027	0.6853483	20	5'UTR	opensea	CRP
cg18317554	-0.0101	0.0055	0.06465	-0.0265235	0.0147277	0.07171498	0.952046	20	5'UTR	opensea	CRP
cg24286200	-0.0092	0.0055	0.09097	0.0416487	0.0260244	0.1095169	0.5423898	11	5'UTR	shelf	CRP
cg02072495	0.008	0.0049	0.1038	0.0225956	0.0140468	0.1077053	0.1799756	20	5'UTR	shore	CRP
cg03957109	-0.0043	0.0051	0.3972	-0.00393512	0.00466857	0.3992855	0.472363	20	Body	opensea	CRP
cg09533293	0	0.0043	0.9915	0	0.0189793	1	0.3449609	20	5'UTR	shore	CRP
cg09785377	-0.0029	0.0046	0.5291	0.00217965	0.00345753	0.528428	0.4535049	20	Body	opensea	CRP
cg13313836	0.0031	0.0044	0.479	-0.00978348	0.0139228	0.4822458	0.5092191	20	5'UTR	shelf	CRP
cg27554954	0.0033	0.0044	0.4563	-0.00353234	0.00471095	0.4533662	0.2415409	20	TSS1500	shore	CRP
cg04432599	0.0072	0.0062	0.2412	0.0195382	0.0169953	0.2502993	0.4595637	20	1stExon	opensea	IL6
cg04970434	-0.0044	0.0067	0.5114	0.0139072	0.0212928	0.5136653	0.7979913	20	3'UTR	opensea	IL6

cg07892449	0.0082	0.0086	0.3428	0.0171049	0.018097	0.3445666	0.5672068	20	1stExon	opensea	IL6
cg11340260	0.0072	0.0062	0.2412	0.0187736	0.0163208	0.2500267	0.3948057	20	TSS200	opensea	IL6
cg11902961	0.0072	0.0062	0.2412	0.0191252	0.0166323	0.2501907	0.4805276	20	TSS1500	opensea	IL6
cg21934015	-0.0026	0.0056	0.6375	-0.00470345	0.0101362	0.6426302	0.8575484	20	TSS1500	opensea	IL6
cg00716083	0.0015	0.0044	0.7387	0.00394806	0.0115859	0.733281	0.9944255	20	TSS200	shore	IL6
cg04910591	-0.0048	0.0046	0.3035	0.018512	0.0179163	0.3014867	0.9147313	20	Body	shore	IL6
cg10749572	0.0015	0.0044	0.7387	0.00487171	0.0142996	0.7333382	0.9887748	20	TSS1500	shore	IL6
cg16975587	-0.0033	0.0047	0.4865	0.0118829	0.0169901	0.4843035	0.8823761	20	Body	island	IL6
cg17527798	0.0015	0.0044	0.7387	0.00572226	0.0168004	0.7334023	0.9972832	20	TSS200	island	IL6
cg25930780	-0.0043	0.0047	0.3647	0.0172672	0.0190257	0.364104	0.8804654	20	Body	island	IL6
cg26307117	0.0015	0.0044	0.7387	0.0035005	0.0102715	0.7332556	0.9991297	20	TSS200	shore	IL6
cg27314002	0.0015	0.0044	0.7387	0.0061565	0.0180781	0.7334418	0.9919545	20	TSS200	shore	IL6
cg03354519	0.001	0.0054	0.8496	-0.00446041	0.0240993	0.8531628	0.7834825	18	TSS1500	island	IL6
cg08715877	-0.0023	0.0049	0.6316	0.0103856	0.022187	0.6397168	0.120912	20	5'UTR	island	IL6
cg08831277	-0.0005	0.0067	0.9411	0.00131458	0.0176161	0.9405142	0.3907022	20	5'UTR	island	IL6
cg19501813	0.0003	0.0054	0.9625	0.000412309	0.0074216	0.9556961	0.3456607	20	TSS200	island	IL6
cg22097342	0.0002	0.0054	0.9707	0.00052498	0.0141746	0.9704557	0.4497479	20	TSS200	island	IL6
cg20025238	0.0048	0.005	0.3412	0.0184061	0.0193622	0.3417976	0.6487891	11	Body	shore	IL6
cg04632671	0.0062	0.0066	3.45E-01	-0.0178084	0.0191242	3.52E-01	1.19E-01	18	TSS1500	island	TNF
cg23514324	-0.0138	0.0047	3.20E-03	-0.0252037	0.00872508	3.87E-03	9.81E-03	20	TSS1500	island	TNF
cg04704294	-0.0026	0.0046	0.5655	-0.00316451	0.0056	0.5720113	0.1934138	20	TSS1500;TSS1500	N_Shore	IL1_beta
cg05694621	0.0096	0.0043	0.02731	0.00967633	0.00434155	0.025829	0.3129468	20	Body;Body;Body		IL1_beta
cg08716655	-0.0026	0.0046	0.5661	-0.00313947	0.00555567	0.5720111	0.2345783	20	TSS1500;TSS1500	N_Shore	IL1_beta

cg09320652	-0.0146	0.0157	0.3545	0.00514509	0.00553736	0.3528065	0.4782879	15	Body;Body;Body		IL1_beta
cg16294013	-0.0026	0.0046	0.5675	-0.00467453	0.00827513	0.5721488	0.1680105	20	TSS200;TSS1500	N_Shore	IL1_beta
cg22336004	-0.0026	0.0046	0.5675	-0.00622435	0.0110239	0.5723284	0.1467602	20	TSS200;TSS1500	N_Shore	IL1_beta
cg22488256	-0.0026	0.0046	0.5655	-0.00477411	0.00845156	0.5721562	0.3604339	20	TSS200;TSS1500	N_Shore	IL1_beta
cg24058805	0.0118	0.0043	0.006508	0.0335739	0.0126139	0.007775852	0.5778297	20	Body;Body;Body		IL1_beta

The SNP instrument for each drug target gene is the SNP with the most significant association with DNA methylation in the blood (top mQTL SNP). The table shows 1. Association of the SNP instrument with DNA methylation expression in the blood (mQTL association) in the McRae et al summary data, where beta is the standard deviation change in gene expression per coded allele; 2. Association of the SNP instrument with major depressive disorder risk in the Wary et al genome-wide association study, where beta is the log odds ratio per coded allele 3. MR association between gene expression and major depressive disorder, where beta is the log odds per 1 standard deviation increase in gene expression; 4. A significant HEIDI p-value (<0.05) indicates that any association between gene expression and outcome may be due to linkage where there are 2 distinct causal variants in linkage disequilibrium (LD). Derivation of the HEIDI p-value can only be done if a sufficient number of SNPs (5 or more SNPs required) are available in the re gion that has 0.05 < LD r-squared < 0.9 with the top mQTL SNP. Note that the MR effects reported in this table are not harmonised based on the direction of effect of DNA methylation on inflammatory factors level in blood but simply report the effect on disease risk per 1SD increase in DNA methylation. Abbreviations: se, standard error; SNP, single nucleotide polymorphism; mQTL, DNA methylation quantitative trait loci; MR, mendelian randomisation; HEIDI, Heterogeneity in dependent instruments.

eTable 10. Validation MR association between drug target gene DNA methylation in blood and MDD (UKB)(Psmr_threshold < 0.000431[0.05/29/4])

Probe ID	Gene	Chromosome	Probe_bp	top_SNP	top_SNP Chromosome	top_SNP base pair	coded allele	other allele	coded allele frequency	eQTL association			
										beta	se	p-value	F-statistic
cg00397479	NEU1	6	31831510	rs3130063	6	31558702	T	C	0.0797546	-0.25912	0.0437725	3.23E-09	35.04
cg19036153	NEU1	6	31830561	rs41267082	6	31830593	C	A	0.0306748	-0.662126	0.0960838	5.53E-12	47.49
cg25560247	NEU1	6	31832173	rs613165	6	31834398	A	G	0.197342	-0.312305	0.0462606	1.47E-11	45.58
cg00445997	TBXAS1	7	139478495	rs141212865	7	139404666	C	A	0.216769	-0.274549	0.0397754	5.11E-12	47.64
cg03604364	TBXAS1	7	139705703	rs2267705	7	139696248	C	T	0.337423	0.305451	0.0346357	1.16E-18	77.77
cg06750564	TBXAS1	7	139637446	rs2284207	7	139640362	C	T	0.366053	-0.193134	0.0351754	4.01E-08	30.15
cg14116596	TBXAS1	7	139528673	rs6952322	7	139524753	A	G	0.44274	-0.411675	0.0314918	4.73E-39	170.89
cg18317554	TBXAS1	7	139498041	rs7459106	7	139439007	G	A	0.216769	0.380794	0.0413404	3.23E-20	84.85
cg24286200	TBXAS1	7	139481268	rs4132077	7	139420225	C	T	0.215746	-0.220895	0.0401575	3.78E-08	30.26
cg02072495	ANXA2	15	60689285	rs11071525	15	60689238	C	G	0.276074	0.354051	0.0376466	5.22E-21	88.45
cg03957109	ANXA2	15	60654693	rs8029375	15	60654671	G	A	0.244376	1.09272	0.030885	3.43E-274	1251.76
cg09533293	ANXA2	15	60689670	rs59233126	15	60691920	A	G	0.474438	0.226562	0.0315143	6.52E-13	51.68
cg09785377	ANXA2	15	60644157	rs35828699	15	60639256	T	C	0.327198	-1.33049	0.019634	0	4592.04
cg13313836	ANXA2	15	60687284	rs12440452	15	60687230	A	C	0.416155	-0.316861	0.0326589	2.95E-22	94.13
cg27554954	ANXA2	15	60691595	rs8033800	15	60689179	T	A	0.419223	-0.934225	0.0276842	1.23E-249	1138.78
cg22431228	CLCNKA	1	16359049	rs1805152	1	16356501	G	A	0.444785	0.891623	0.028266	2.17E-218	995.02
cg10589539	NR1I2	3	119514395	rs1357459	3	119514556	C	T	0.259714	0.422697	0.034599	2.52E-34	149.26
cg16322565	NR1I2	3	119526221	rs6772249	3	119908479	T	C	0.014315	-1.00331	0.089703	4.84E-29	125.10
cg08715877	ANXA5	4	122618117	rs11098635	4	122586056	A	G	0.244376	-0.22146	0.035092	2.77E-10	39.83

cg04632671	PPARG	3	12329826	rs11709077	3	12336507	A	G	0.118609	-0.348151	0.049291	1.63E-12	49.89
cg25929976	PPARG	3	12328656	rs62242116	3	12316326	A	G	0.072597	-0.350555	0.055852	3.46E-10	39.39
cg04704294	PDE4B	1	66257822	rs11208743	1	66242820	T	C	0.327198	0.821612	0.0306521	2.87E-158	718.48
cg05694621	PDE4B	1	66767231	rs6683977	1	66769100	G	C	0.436605	0.992111	0.0259091	0	1466.28
cg08716655	PDE4B	1	66257732	rs12401401	1	66231308	A	G	0.327198	0.828167	0.0308486	9.35E-159	720.72
cg09320652	PDE4B	1	66708864	rs74531536	1	66711918	G	A	0.0245399	-2.83766	0.12481	1.98E-114	516.92
cg16294013	PDE4B	1	66258022	rs12034009	1	66255225	C	T	0.327198	0.556205	0.0335251	8.13E-62	275.25
cg22336004	PDE4B	1	66258035	rs12034009	1	66255225	C	T	0.327198	0.417714	0.033843	5.33E-35	152.34
cg22488256	PDE4B	1	66258046	rs11208743	1	66242820	T	C	0.327198	0.544605	0.0333677	6.96E-60	266.39
cg24058805	PDE4B	1	66777579	rs2180336	1	66777996	T	C	0.43865	0.351464	0.0321416	7.85E-28	119.57

eTable 10. Validation MR association between drug target gene DNA methylation in blood and MDD (UKB)(Psmr_threshold < 0.000431[0.05/29/4]) (Continued)

Probe ID	GWAS association			MR association (log odds per 1SD increase in expression)			HEIDI Test		DNAm feature		Phenotype
	beta	se	p-value	beta	se	p-value	p-value	Number of SNPs	feature	cgi	
cg00397479	-0.0078968	0.0017308	5.06E-06	-0.0304755	0.00843325	0.000301823	0.1486754	20	TSS1500	shore	CRP
cg19036153	-0.0053441	0.0034226	0.1184268	0.00807112	0.00530014	0.1278053	0.2120674	20	5'UTR	island	CRP
cg25560247	-0.00099171	0.0016414	0.5457202	0.00317545	0.00527676	0.5473204	0.07043052	20	TSS1500	shore	CRP
cg00445997	-0.0019473	0.0014962	0.193099	0.00709273	0.0055457	0.200911	0.6359128	20	5'UTR	island	CRP
cg03604364	-0.00076742	0.0012784	0.5483148	-0.00251242	0.00419497	0.549232	0.2744112	20	Body	opensea	CRP
cg06750564	0.0023373	0.0012464	0.06074353	-0.012102	0.00681957	0.07596496	0.9914534	15	Body	opensea	CRP
cg14116596	-4.04E-05	0.0011804	0.9726868	9.82E-05	0.00286732	0.9726858	0.9778199	20	5'UTR	opensea	CRP

cg18317554	-0.0021046	0.0014921	0.1583981	-0.00552687	0.00396406	0.163244	0.5459687	20	5'UTR	opensea	CRP
cg24286200	-0.0021042	0.0014983	0.1602065	0.00952578	0.00700043	0.173595	0.5850584	11	5'UTR	shelf	CRP
cg02072495	0.002849	0.0013508	0.03493011	0.00804686	0.00391004	0.03958993	0.5429054	20	5'UTR	shore	CRP
cg03957109	-0.0011969	0.0013832	0.3868477	-0.00109534	0.00126621	0.3870094	0.393443	20	Body	opensea	CRP
cg09533293	-0.0012467	0.0011822	0.2916285	-0.00550269	0.00527383	0.296766	0.4596004	20	5'UTR	shore	CRP
cg09785377	0.00037281	0.0012466	0.7648918	-0.000280206	0.00093696	0.7648954	0.6957741	20	Body	opensea	CRP
cg13313836	0.0016208	0.0012102	0.1804845	-0.00511518	0.00385556	0.1846072	0.9421849	20	5'UTR	shelf	CRP
cg27554954	0.0016556	0.0012046	0.1693246	-0.00177216	0.00129048	0.1696716	0.319155	20	TSS1500	shore	CRP
cg22431228	0.000489	0.001187	0.680299	0.000548		0.680311	0.021877	20	Body	opensea	IL6
cg10589539	0.000338	0.001279	0.79179	0.000799	0.003027	0.791829	0.460852	20	5'UTR	opensea	IL6
cg16322565	-0.006672	0.003228	0.038708	0.00665	0.003272	0.042073	0.611273	20	Body	shelf	IL6
cg08715877	-0.000478	0.00133	0.719217	0.002159	0.006014	0.719649	0.083115	20	TSS1500	island	IL6
cg04632671	0.001137	0.001815	0.531104	-0.003266	0.005235	0.532719	0.058855	16	TSS1500	island	TNF
cg25929976	0.006001	0.002008	0.002796	-0.01712	0.006343	0.006958	0.016344	20	TSS1500	shore	TNF
cg04704294	0.00053469	0.0012589	0.6710425	0.000650782	0.00153242	0.6710739	0.4250055	20	TSS1500;TSS1500	N_Shore	IL1_beta
cg05694621	0.0010037	0.0011875	0.3979789	0.00101168	0.00119723	0.3981023	0.1835772	20	Body;Body;Body		IL1_beta
cg08716655	0.00054414	0.001259	0.6655796	0.000657042	0.00152042	0.6656366	0.3679555	20	TSS1500;TSS1500	N_Shore	IL1_beta
cg09320652	-0.0089143	0.0042437	0.03567796	0.00314143	0.00150186	0.03646646	0.2649465	15	Body;Body;Body		IL1_beta
cg16294013	0.0005295	0.0012591	0.6740933	0.000951987	0.00226446	0.6741908	0.3343771	20	TSS200;TSS1500	N_Shore	IL1_beta
cg22336004	0.0005295	0.0012591	0.6740933	0.00126761	0.00301601	0.6742703	0.2322378	20	TSS200;TSS1500	N_Shore	IL1_beta
cg22488256	0.00053469	0.0012589	0.6710425	0.000981795	0.00231237	0.6711398	0.332388	20	TSS200;TSS1500	N_Shore	IL1_beta
cg24058805	0.0033391	0.0011891	0.004983105	0.00950056	0.00349306	0.006531367	0.4361091	20	Body;Body;Body		IL1_beta

The SNP instrument for each drug target gene is the SNP with the most significant association with DNA methylation in the

blood (top mQTL SNP). The table shows 1. Association of the SNP instrument with DNA methylation expression in the blood (mQTL association) in the Hannon et al summary data, where beta is the standard deviation change in gene expression per coded allele; 2. Association of the SNP instrument with major depression disorder risk (UKB) in the Howard et al genome-wide association study, where beta is the log odds ratio per coded allele 3. MR association between gene expression and major depressive disorder, where beta is the log odds per 1 standard deviation increase in gene expression; 4. A significant HEIDI p-value (<0.05) indicates that any association between gene expression and outcome may be due to linkage where there are 2 distinct causal variants in linkage disequilibrium (LD). Derivation of the HEIDI p-value can only be done if a sufficient number of SNPs (5 or more SNPs required) are available in the region that has 0.05 < LD r-squared < 0.9 with the top mQTL SNP. Note that the MR effects reported in this table are not harmonised based on the direction of effect of DNA methylation on inflammatory factors level in blood but simply report the effect on disease risk per 1SD increase in DNA methylation. Abbreviations: se, standard error; SNP, single nucleotide polymorphism; mQTL, DNA methylation quantitative trait loci; MR, mendelian randomisation; HEIDI, Heterogeneity in dependent instruments.

eTable 11. MR association between NEU1 DNA methylation in blood and gene expression (Psmr_threshold < 0.05 & PHEIDI_threshold > 0.05)

Functional Genes		Methylation					Gene expression		Methylation - MDD				
Gene	Chr	Me_probe	Me_bp	feature	cgi	feat.cgi	Probe ID	Probe bp	beta_SMR	se_SMR	p_SMR	p_HEIDI	Pass_HEIDI
NEU1	6	cg00397479	31831510	TSS1500	shore	TSS1500-shore	ENSG00000204386	31828059	-0.120334	0.0295694	4.71E-05	0.2524661	20

eTable 11. MR association between NEU1 DNA methylation in blood and gene expression (Psmr_threshold < 0.05 & PHEIDI_threshold > 0.05) (Continued)

Methylation - Gene expression					Gene expression - MDD					Top SNP for methylation - MDD				Top SNP for methylation - gene expression			
beta_SMR	se_SMR	p_SMR	p_HEIDI	Pass_HEIDI	beta_SMR	se_SMR	p_SMR	p_HEIDI	Pass_HEIDI	SNP	SNP_bp	A1	A2	SNP	SNP_bp	A1	A2
-0.518603	0.101419	3.16E-07	2.25E-01	20	0.215192	0.0472951	5.36E-06	0.2945077	20	rs693906	31835164	C	G	rs433061	32014828	G	A

eTable 11. MR association between NEU1 DNA methylation in blood and gene expression (Psmr_threshold < 0.05 & PHEIDI_threshold > 0.05) (Continued)

Top SNP for gene expression - MDD				r2 between top SNPs			D' between top SNPs		
SNP	SNP_bp	A1	A2	r2_12	r2_13	r2_23	D'_12	D'_13	D'_23
rs367364	32019946	T	C	0.6227	0.3491	0.5948	0.9334	0.6476	1

Methylation-gene expression SMR analysis was performed between the 1 DNAm and 1 gene expression probes that both passed the SMR & HEIDI test. For each row, the r2 between any 2 of the 3 tops SNPs was calculated using "LDlink" package. r2_12 refers to r2 between SNPs for methylation-MDD and SNPs for methylation-gene expression; r2_13 refers to r2 between SNPs for methylation-MDD and SNPs for gene expression-MDD; r2_23 refers to r2 between SNPs for methylation-gene expression, and SNPs for gene expression-MDD.

eTable 12. Validation of MR association analysis, gene NEU1 DNA methylation in blood and gene expression

Functional Genes		Methylation					Gene expression		Methylation - MDD					Methylation - Gene expression				
Gene	Chr	Me_probe	Me_bp	feature	cgi	feat.cgi	Exp_probe	Exp_bp	b_SMR	SE_SMR	P_SMR	p_HEIDI	Pass_HEIDI	b_SMR	SE_SMR	P_SMR	p_HEIDI	Pass_HEIDI
NEU1	1	cg00397479	31831510	TSS1500	shore	TSS1500_shore	ILMN_1763144	31827313	-0.031	0.008	0.000301823	0.1486754	20	-0.841062	0.198467	2.26E-05	5.32E-01	20

eTable 12. Validation of MR association analysis, gene NEU1 DNA methylation in blood and gene expression (Continued)

Methylation - MDD					Methylation - Gene expression					Gene expression - MDD				
b_SMR	SE_SMR	P_SMR	p_HEIDI	Pass_HEIDI	b_SMR	SE_SMR	P_SMR	p_HEIDI	Pass_HEIDI	b_SMR	SE_SMR	P_SMR	P_HEIDI	Pass_HEIDI
-0.031	0.008	0.000301823	0.1486754	20	-0.841062	0.198467	2.26E-05	5.32E-01	20	0.0336906	0.0097082	0.000519834	0.6963878	20

eTable 12. Validation of MR association analysis, gene NEU1 DNA methylation in blood and gene expression (Continued)

Top SNP for methylation - MDD				Top SNP for methylation - gene expression				Top SNP for gene expression - MDD				r2 between top SNPs		
SNP	SNP_bp	A1	A2	SNP	SNP_bp	A1	A2	SNP	SNP_bp	A1	A2	r2_12	r2_13	r2_23
rs3130063	31558702	T	C	rs433061	32014828	A	G	rs3130063	31558702	T	C	0.4577	1	0.4577

Methylation-gene expression SMR analysis was performed between the 1 DNAm and 1 gene expression probes that both passed the SMR & HEIDI test. For each row, the r2 between any 2 of the 3 tops SNPs was calculated using "LDlink" package. r2_12 refers to r2 between SNPs for methylation-MDD and SNPs for methylation-gene expression; r2_13 refers to r2 between SNPs for methylation-MDD and SNPs for gene expression-MDD; r2_23 refers to r2 between SNPs for methylation-gene expression, and SNPs for gene expression-MDD.

MR Power Calculation

eTable 13. Association Between NEU1 Brain Expression and MDD Risk(Psmr_threshold < 0.0035[0.05/14])

Tissue	Top-associated SNP	A1freq	F-statistic	r-square	eQTL Samplesize	Ratio cases:controls	odds ratio with 80% power
Brain substantia nigra (n = 139)	rs2507967	0.27	12.96	0.000652654	139	1.84	1.469
Brain amygdala (n = 152)	rs13197153	0.02	12.96	0.000551109	152	1.84	1.524
Brain spinal cord cervical C1 (n = 159)	rs17421624	0.33	12.96	0.001086745	159	1.84	1.346
Brain anterior cingulate cortex BA24 (n = 176)	rs116212553	0.08	12.96	1.00078E-06	176	1.84	>100
Brain hippocampus (n = 197)	rs13208918	0.01	12.96	1.21474E-05	197	1.84	16.346
Brain hypothalamus (n = 202)	rs150934595	0.03	12.96	0.00016831	202	1.84	2.125
Brain putamen basal ganglia (n = 205)	rs115333512	0.05	12.96	0.000294037	205	1.84	1.781
Brain frontal cortex BA9 (n = 209)	rs9274407	0.81	12.96	3.68609E-05	209	1.84	4.789
Brain cerebellar hemisphere (n = 215)	rs115277907	0.01	12.96	0.000118418	215	1.84	2.481
Brain cerebellum (n = 241)	rs116198852	0.06	12.96	7.22474E-05	241	1.84	3.155
Brain caudate basal ganglia (n = 246)	rs2070121	0.08	12.96	0.000453777	246	1.84	1.595
Brain nucleus accumbens basal ganglia (n = 246)	rs116654558	0.05	12.96	0.000238457	246	1.84	1.895
Brain cortex (n = 255)	rs2763977	0.11	12.96	0.000345913	255	1.84	1.695
PsychENCODE prefrontal cortex (n=1387)	rs116198852	0.21	12.96	0.000245299	1387	1.84	1.653

Expression quantitative trait loci data were queried for 13 different brain regions from GTEX v8.

Associations are statistically significant after correcting for 14 tests or did not pass the multiple testing correction [multiple testing correction (p<0.0035)]. BA indicates Brodmann area. Genes for which there was an association with nominal significance as well as passed the multiple testing correlation are marked by a sigle asterisk(*).

eTable 14. Association between NEU1 eQTL SNP (rs367364) in blood with expression of other nearby genes (P_threshold < 0.05)

Gene	Gene Chromosome	Gene start position (hg19)	eQTL SNP	SNP Chromosome	SNP position (hg19	base pair distance of gene from SNP	coded allele	other allele	coded allele frequency	eQTL association		
										beta	se	p-value
HLA-C	6	31238216	rs367364	6	32019946	781730	T	C	0.134439	0.345158	0.013015	5.70E-155
HLA-DQB1	6	32631702	rs367364	6	32019946	-611756	T	C	0.134439	-0.249885	0.0133342	2.33E-78
HLA-DRB5	6	32491592	rs367364	6	32019946	-471646	T	C	0.134439	-0.169764	0.0130982	2.04E-38
SKIV2L	6	31932194	rs367364	6	32019946	87752	T	C	0.134439	-0.197613	0.0177164	6.83E-29
ATF6B	6	32080991	rs367364	6	32019946	-61045	T	C	0.134439	0.221299	0.0223684	4.45E-23
GPANK1	6	31631533	rs367364	6	32019946	388413	T	C	0.134439	-0.220067	0.0223696	7.74E-23
SNHG32	6	31804963	rs367364	6	32019946	214983	T	C	0.134439	-0.167963	0.017739	2.84E-21
NEU1	6	31828059	rs367364	6	32019946	191887	T	C	0.134439	-0.146845	0.0177525	1.32E-16
CCHCR1	6	31118115	rs367364	6	32019946	901831	T	C	0.134439	0.135244	0.0177602	2.64E-14
PSMB9	6	32819637	rs367364	6	32019946	-799691	T	C	0.134439	0.129021	0.0177632	3.77E-13
APOM	6	31623090	rs367364	6	32019946	396856	T	C	0.134439	-0.127942	0.0177632	5.91E-13
LY6G5B	6	31639748	rs367364	6	32019946	380198	T	C	0.134439	0.115767	0.0177704	7.29E-11
HLA-DRB6	6	32524144	rs367364	6	32019946	-504198	T	C	0.134439	-0.0771111	0.0120317	1.46E-10
HLA-DQA1	6	32712055	rs367364	6	32019946	-692109	T	C	0.134439	-0.149296	0.0237611	3.32E-10
PSMB8-AS1	6	32813067	rs367364	6	32019946	-793121	T	C	0.134439	-0.171568	0.0298038	8.58E-09
C2	6	31889505	rs367364	6	32019946	130441	T	C	0.134439	0.0990854	0.0177776	2.50E-08
HCP5	6	31406881	rs367364	6	32019946	613065	T	C	0.134439	-0.0968842	0.0177782	5.05E-08
HCG22	6	31024447	rs367364	6	32019946	995499	T	C	0.134439	-0.167671	0.0309129	5.83E-08

FKBPL	6	32097276	rs367364	6	32019946	-77330	T	C	0.134439	-0.0937812	0.0177791	1.33E-07
RNF5	6	32149030	rs367364	6	32019946	-129084	T	C	0.134439	-0.124154	0.0237798	1.78E-07
DXO	6	31938828	rs367364	6	32019946	81118	T	C	0.134439	-0.107854	0.0224658	1.58E-06
TNXB	6	32046021	rs367364	6	32019946	-26075	T	C	0.134439	-0.126151	0.0273493	3.98E-06
TAP2	6	32798083	rs367364	6	32019946	-778137	T	C	0.134439	-0.0815054	0.0177835	4.58E-06
PRRC2A	6	31597022	rs367364	6	32019946	422924	T	C	0.134439	-0.0994916	0.0224698	9.52E-06
HLA-B	6	31323307	rs367364	6	32019946	696639	T	C	0.134439	-0.104737	0.023792	1.07E-05
EGFL8	6	32134209	rs367364	6	32019946	-114263	T	C	0.134439	0.0773837	0.0177853	1.36E-05
PPT2	6	32126336	rs367364	6	32019946	-106390	T	C	0.134439	0.09594	0.0224737	1.96E-05
MICA	6	31377224	rs367364	6	32019946	642722	T	C	0.134439	0.0866759	0.0224782	0.00011526
BAG6	6	31613643	rs367364	6	32019946	406303	T	C	0.134439	0.0750785	0.0224826	0.0008396
HLA-DOB	6	32782682	rs367364	6	32019946	-762736	T	C	0.134439	0.0614802	0.018426	0.000848101
AIF1	6	31583879	rs367364	6	32019946	436067	T	C	0.134439	-0.0592207	0.0177904	0.00087221
TNF	6	31544728	rs367364	6	32019946	475218	T	C	0.134439	-0.0568493	0.0177899	0.0013954
PRRT1	6	32119143	rs367364	6	32019946	-99197	T	C	0.134439	0.0559799	0.0177917	0.00165294
NFKBIL1	6	31520626	rs367364	6	32019946	499320	T	C	0.134439	0.0550679	0.0177925	0.00196813
HSPA1A	6	31784504	rs367364	6	32019946	235442	T	C	0.134439	0.0690019	0.022485	0.0021492
CSNK2B	6	31635566	rs367364	6	32019946	384380	T	C	0.134439	0.0494456	0.0177939	0.00545601
DDX39B	6	31504110	rs367364	6	32019946	515836	T	C	0.134439	-0.0613935	0.0238125	0.00993165
NELFE	6	31923375	rs367364	6	32019946	96571	T	C	0.134439	-0.0606821	0.0238118	0.0108218
POU5F1	6	31139380	rs367364	6	32019946	880566	T	C	0.134439	-0.0677038	0.027386	0.0134284
HLA-DMA	6	32926630	rs367364	6	32019946	-906684	T	C	0.134439	0.0439657	0.0184296	0.0170513
HLA-DOA	6	32974672	rs367364	6	32019946	-954726	T	C	0.134439	0.0415415	0.0184301	0.0241962

LSM2	6	31769967	rs367364	6	32019946	249979	T	C	0.134439	-0.037136	0.017794	0.0368881
ATP6V1G2	6	31514221	rs367364	6	32019946	505725	T	C	0.134439	0.0370666	0.0177948	0.0372512

Abbreviations: eQTL, expression quantitative trait loci.

eTable 15. Causal estimates from different MR methods for the association of inflammantory factors level with Major depression disorder

CRP levels with Major depression disorder					
method	number of SNPs	beta	OR (95% CI)	se	p-value
GSMR	56	0.0097	1.01 (0.987 - 1.033)	0.0115	0.40
MR Egger	64	-0.0145	0.99 (0.947 - 1.025)	0.0201	0.47
Weighted median	64	-0.0194	0.98 (0.949 - 1.012)	0.0164	0.24
Inverse variance weighted	64	0.0023	1.002(0.976 - 1.029)	0.0133	0.86
Simple mode	64	-0.0250	0.98 (0.909 - 1.046)	0.0346	0.48
Weighted mode	64	-0.0127	0.99 (0.959 - 1.0161)	0.0149	0.40
IL-6 levels with Major depression disorder					
method	number of SNPs	beta	OR (95% CI)	se	p-value
GSMR	9	-0.0255	0.97(0.942 - 1.008)	0.0174	0.14
MR Egger	10	-0.0046	0.99(0.930 - 1.065)	0.0342	0.90
Weighted median	10	0.0175	1.02 (0.973 - 1.064)	0.0227	0.44
Inverse variance weighted	10	0.0203	1.02(0.984 - 1.057)	0.0182	0.26
Simple mode	10	0.0397	1.04(0.960 - 1.127)	0.0409	0.36
Weighted mode	10	-0.0063	0.99(0.928 - 1.063)	0.0346	0.86
TNF with Major depression disorder					
method	number of SNPs	beta	OR (95% CI)	se	p-value
GSMR	5	0.0071	1.007(0.987 - 1.027)	0.0100	0.48
MR Egger	5	0.0004	1.000(0.904 - 1.108)	0.0520	0.99
Weighted median	5	-0.0061	0.994(0.971 - 1.018)	0.0120	0.61

Inverse variance weighted	5	-0.0018	0.998(0.974 - 1.023)	0.0127	0.88
Simple mode	5	-0.0212	0.979(0.938 - 1.021)	0.0217	0.38
Weighted mode	5	-0.0184	0.982(0.947 - 1.017)	0.0183	0.37

Abbreviations: CRP, C-reactive protein level; IL-6, Interleukin 6; TNF, Tumor necrosis factor; se, standard error; SNP, single nucleotide polymorphism; OR odds ratio; CI confidence intervals

MR Power Calculation

eTable 16. Association Between NEU1 Blood Gene Expression and MDD risk(Psmr_threshold < 0.00333[0.05/16])

Gene	SNP	F-statistic	r-square	Samplesize	Ratio cases:controls	Causal effect with 80% power	Phenotype	OR
CASP1	rs2409065	1037.40	2.68254E-06	322580	1.84	>100	CRP	0.9983229
CCND1	rs1960217	411.42	0.000480078	322580	1.84	1.605	CRP	1.0308238
NEU1**	rs367364	68.42	0.009201122	322580	1.84	1.114	CRP	1.2401
PTGDR2	rs530963	162.12	0.000432974	322580	1.84	1.644	CRP	1.0467657
ACAT1	rs4550189	1043.36	1.29701E-05	322580	1.84	>100	CRP	1.0023864
TBXAS1	rs2240395	1223.48	0.002131534	322580	1.84	1.251	CRP	1.0423447
NR3C2	rs6817545	88.12	0.000338822	322580	1.84	1.751	CRP	0.9416893
ANXA2	rs62004990	937.17	0.000754412	322580	1.84	1.459	CRP	1.0281378
GP1BA	rs67059207	93.21	0.001737147	322580	1.84	1.265	IL6	0.983673
LTF	rs4683221	50.01	0.001746452	322580	1.84	1.266	IL6	1.0084468
NR1I2	rs3732357	34.54	0.001396798	322580	1.84	1.299	IL6	1.1428133
ANXA5	rs62320625	8054.82	0.001228532	322580	1.84	1.325	IL6	1.0002454
SMO	rs7798321	78.45	0.001475081	322580	1.84	1.295	IL6	0.9136192
HSPA5	rs2416962	251.27	0.002640165	322580	1.84	1.213	TNF	0.9102227
PPARG	rs1699346	1012.10	1.0248E-05	322580	1.84	20.989	TNF	0.996841
LTB4R	rs3181256	311.46	0.00021155	322580	1.84	1.967	TNF	0.9745339

For 1 out of the 16 we have 80% power to detect (at least 5% significance level) a smaller causal effect of gene expression on major depression disorder than that observed for NEU1 (OR = 1.240). Genes for which there was an association with at least nominal significance are marked by a single asterisk (*), while those that passed multiple testing correlation (P < 0.003125) are marked by double asterisks (*).
