# Supplementary material for: Non-steroidal anti-inflammatory drug target gene associations with major depressive disorders: a Mendelian randomisation study integrating GWAS, eQTL and mQTL Data
Source: Pharmacogenomics J. 2023 Mar 25;23(4):95–104. doi: 10.1038/s41397-023-00302-1 (PMC10382318; doi:10.1038/s41397-023-00302-1)
Supplement: Supplementary file 2 — Supplementary figures [file 41397_2023_302_MOESM2_ESM.docx]

**Supplementary Figures**

eFigure1. Validation analysis of association drug target gene expression in blood with MDD (UKB) risk

eFigure2. Possible explanations for observed association in mendelian randomization (MR) analysis between *NEU1* gene expression and major depressive disorder

eFigure 3. *NEU1* differential gene expression and DNA methylation between depressive patients and healthy controls blood samples

eFigure 4. *NEU1* gene expression in different tissues


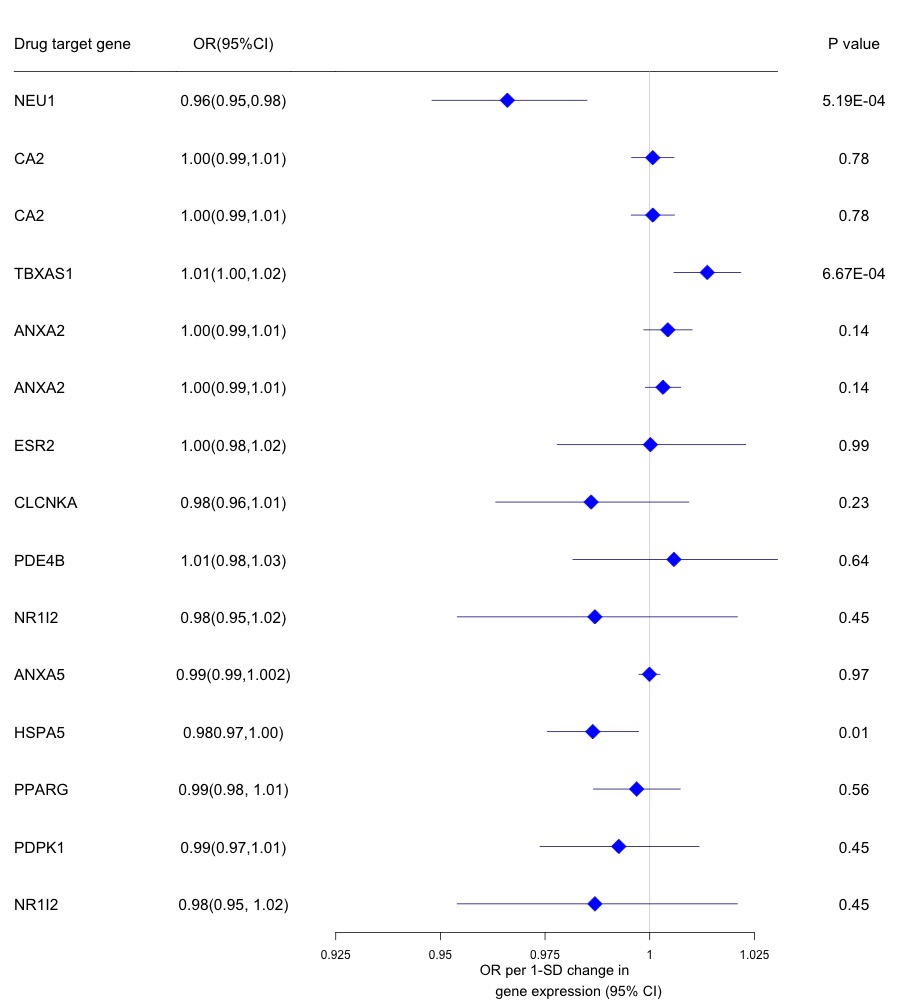


**eFigure 1. Validation of association drug target gene expression in blood with MDD (UKB) risk**

Forest plot of the association between a 1-SD change in expression of 16 inflammatory factor levels lowering drug target genes in blood with risk for major depressive disorder (UKB). Data are represented as odds ratios (ORs) with 95% CI (error bars). The direction of gene expression change reflects the inflammatory factor levels lowering association. Therefore, an OR of greater than 1.00 suggests an decreased risk of major depressive disorder associated with NSAIDs treatment. Associations are statistically significant after correcting for multiple testing (16 genes x 3 inflammatory factors) and have a heterogeneity in dependent instrument (HEIDI) P >= 0.05, statistically significant after correcting for multiple testing but have a HEIDI P < 0.05 (indicating association likely due to linkage) or did not pass the multiple testing correction. SMR indicates summary-based mendelian randomization.


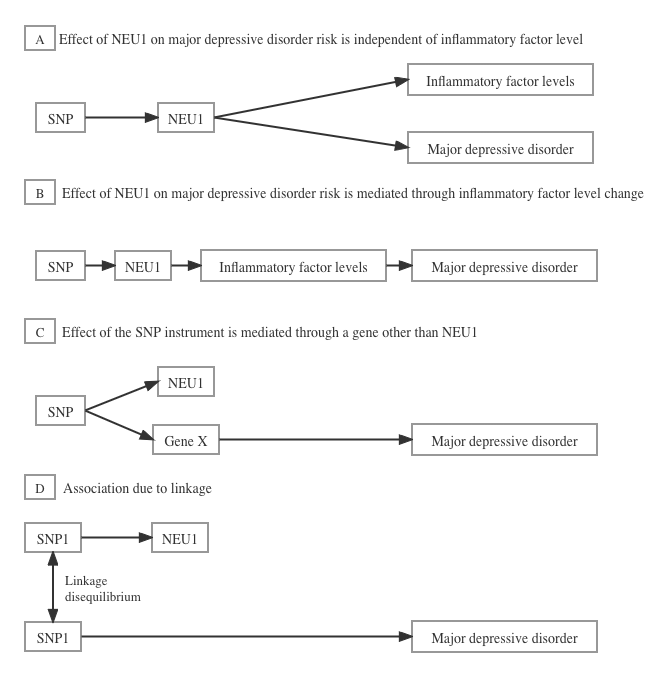


**eFigure 2. Possible explanations for observed association in mendelian randomization (MR) analysis between *NEU1* gene expression and major depressive disorder**

(A) NEU1 has an effect on major depressive disorder (MDD) that is independent of its effect on inflammatory factor level, assessed by MR analysis using inflammatory factor level as exposure. (B) The effect of *NEU1* on MDD risk is mediated through inflammatory factor level change, assessed by MR analysis using inflammatory factor level as exposure. (C) The *NEU1* expression quantitative trait loci (eQTL) single-nucleotide variant (SNP) instrument is also associated with expression of another gene. MDD risk is mediated through this gene rather than through NEU1 and assessed by MR and colocalization analysis of other genes associated with the *NEU1* SNP instrument. (D) Linkage association, whereby 2 SNPs are in linkage disequilibrium. One SNP affects *NEU1* expression whereas the other affects MDD risk through its effect on expression of another gene. The association is assessed by heterogeneity in dependent instrument test and colocalization analysis.


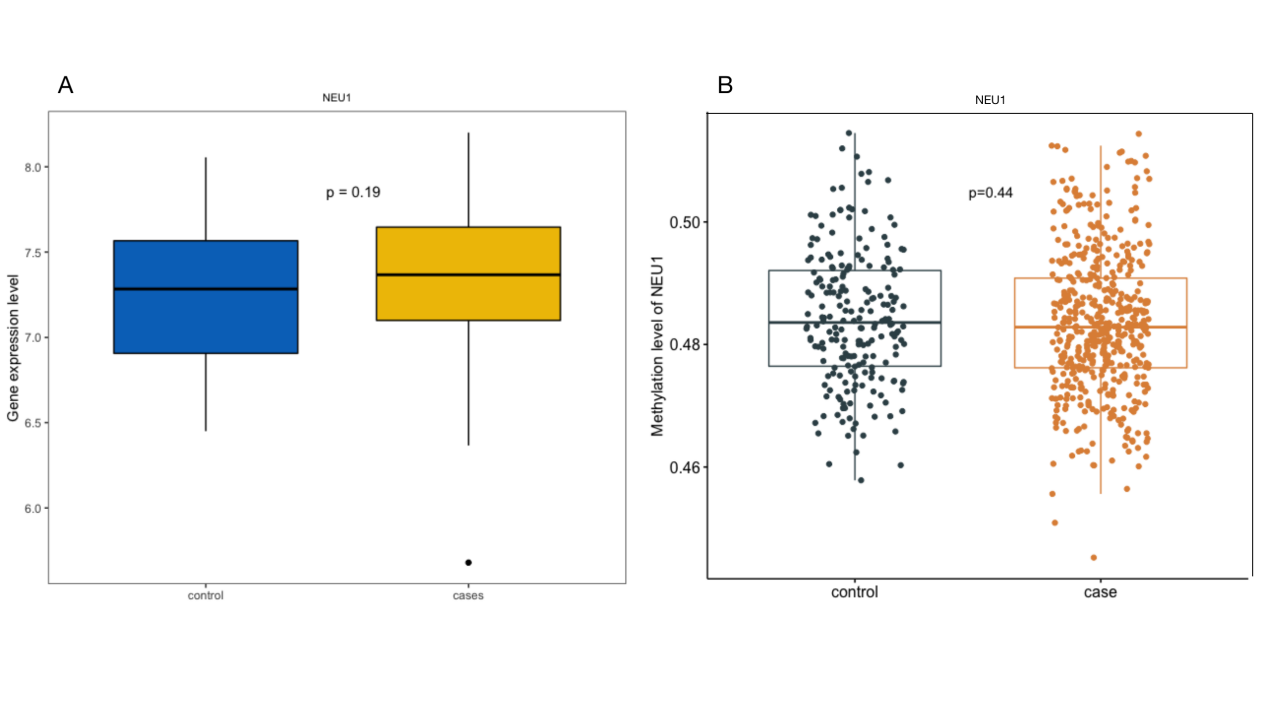


**eFigure 3. *NEU1* differential gene expression and DNA methylation between depressive patients and healthy controls (blood samples)**

1. The gene expression levels of *NEU1* gene was insignificant in GSE98793 dataset. (B) The DNA methylation levels of *NEU1* gene was insignificant in GSE125105 dataset.


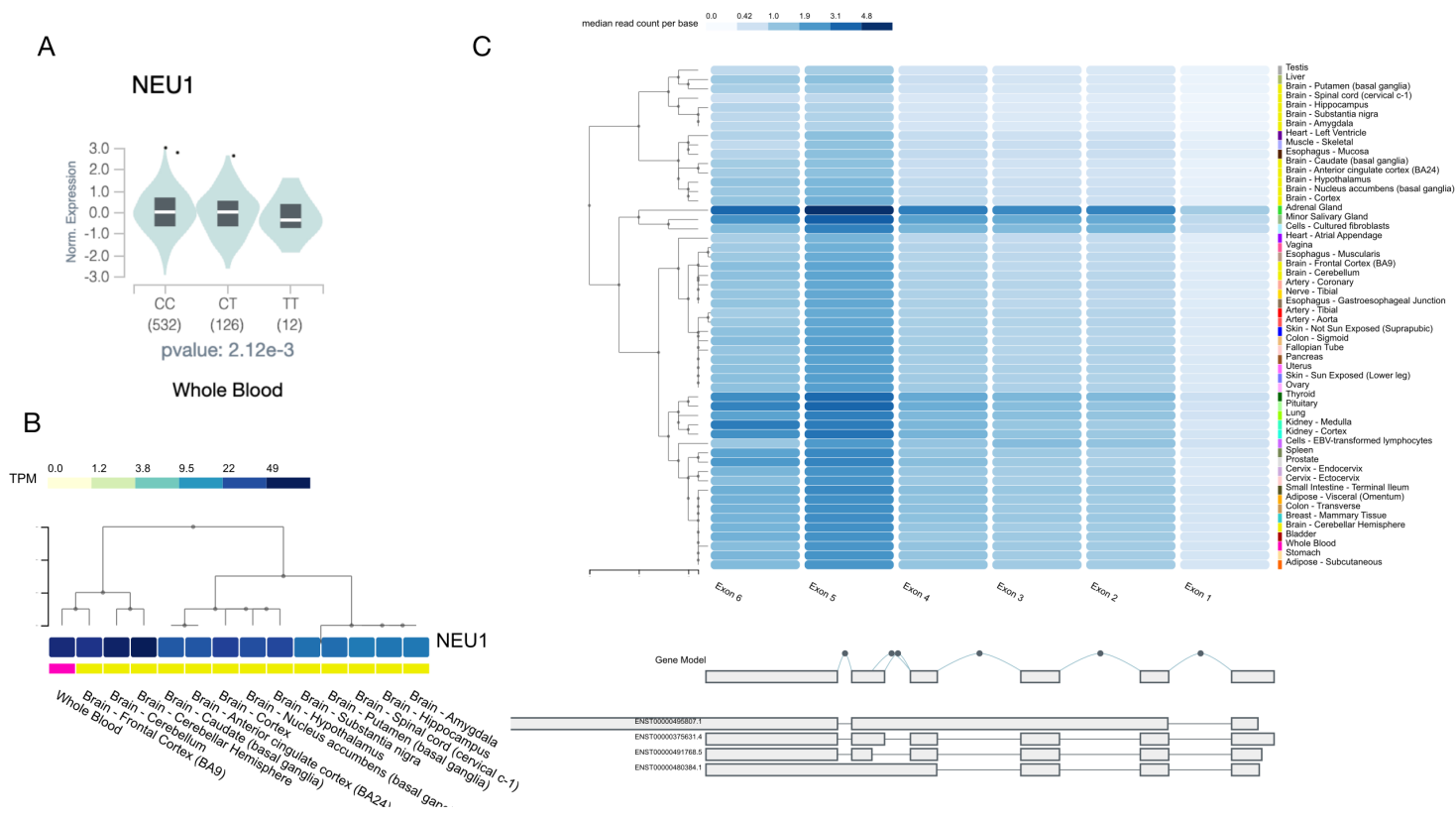


**eFigure 4. *NEU1* gene expression in different tissues.**

(A) eQTL analysis showed the association of expression-related SNP (rs2242664, risk allele T) and the expression of *NEU1* in whole blood from GTEx (*P* = 2.12 × 10^-3^). (B) The expression of *NEU1* in 12 different brain regions and whole blood from GTEx. (C) The different expression of *NEU1* in 54 tissues from GTEx.
