# Supplementary material for: Non-steroidal anti-inflammatory drug target gene associations with major depressive disorders: a Mendelian randomisation study integrating GWAS, eQTL and mQTL Data
Source: Pharmacogenomics J. 2023 Mar 25;23(4):95–104. doi: 10.1038/s41397-023-00302-1 (PMC10382318; doi:10.1038/s41397-023-00302-1)
Supplement: Supplementary file 3 — Supplementary methods [file 41397_2023_302_MOESM3_ESM.docx]

**Supplementary Methods**

**SMR analysis**

We used the summary-based MR (SMR) method, version 1.02, to perform 2-sample MR analysis. Briefly, let y be the outcome of interest, x be gene expression or DNA methylation (exposure), g be a genetic instrument, *b_gx_* be the effect of g on x (estimated by eQTL/mQTL studies), and *b_gy_* be the effect g on the y (estimated by GWAS). The effect of exposure x on outcome y (*b_xy_*), free of any non-genetic confounders, is defined as b_gy_/b_gx_ (Wald ratio).

The SMR approach selects a single most significantly associated eQTL/mQTL SNP (located near the target gene i.e. cis-eQTL SNP) as instrument. SMR requires a reference dataset from which to estimate LD between SNPs. For this purpose, we use 486 unrelated individuals of European ancestry from the 1000 Genome project was used. There to be no overlap between exposure and outcome data, ifelse, SMR test statistics still have been shown to be robust to even complete sample overlap.

**Assessing association due to linkage**

The SMR tool also implements the heterogeneity in dependent instruments (HEIDI) test to assess if the observed association between gene expression and outcome is due to a linkage scenario, where rather than the SNP affecting disease via gene expression regulation, the SNP that influences expression is in linkage disequilibrium (LD) with another SNP that independently influences the outcome. If gene expression and a trait share the same causal variant, the *b_xy_* values calculated for any SNPs in LD (using the default value of *r^2^* > 0.05 and also *r^2^* < 0.9 to avoid issues of collinearity) with the causal variant should be identical. Therefore, testing against this null hypothesis of a single causal variant is equivalent to testing for heterogeneity in the *b_xy_* values estimated for the SNPs in the cis-eQTL region. Since heterogeneity estimates may not be robust if using only a small number of SNPs, the HEIDI test required a minimum of 5 SNPs for estimating heterogeneity. A HEIDI test *P* < 0.05 was considered to indicate heterogeneity in *bxy* values, suggesting that association between gene expression and outcome is most likely to be due to a linkage scenario.

**Weak Instrumental Bias in MR analyses**

The *F* statistic from the regression of the exposure (gene expression or DNAm) on the instrument (eQTL or mQTL SNP) was usually quoted in single sample MR studies as a measure of the strength of an instrument. *F* statistics for SNP *j* could be approximated as $\boldsymbol{F}_{\boldsymbol{j}}\boldsymbol{=}\frac{\hat{\boldsymbol{\gamma}_{\boldsymbol{j}}^{\boldsymbol{2}}}}{\boldsymbol{\sigma}_{\boldsymbol{x}_{\boldsymbol{j}}}^{\boldsymbol{2}}}$where $\hat{\gamma_{j}}$ is the SNP exposure association and $\sigma_{x_{j}}$ is the standard error of the SNP-exposure association.
